# Integration and impact of pharmacists in general practice internationally: A rapid review

**DOI:** 10.1177/13558196231179831

**Published:** 2023-06-17

**Authors:** Georgios Dimitrios Karampatakis, Nilesh Patel, Graham Stretch, Kath Ryan

**Affiliations:** 1 Postdoctoral Researcher-Health Services, Centre for Primary Care, 105714 Wolfson Institute of Population Health, Barts and The London School of Medicine and Dentistry, Queen Mary University of London, London, UK; 2 Associate Professor in Pharmacy, RinggoldId: 14304 University of Reading School of Pharmacy, Reading, UK; 3 Lead Pharmacist, Ealing GP Federation, London, UK; 4 Professor Emerita, RinggoldId: 14304 University of Reading School of Pharmacy, Reading, UK

**Keywords:** Pharmacists in general practice, integration, impact

## Abstract

**Objective:**

English general practices have been facing ongoing pressures, arising from complicated health care needs and the recent pandemic. To overcome these pressures and reduce the workload of general practitioners, there have been extensive attempts to integrate pharmacists into general practices. A number of literature reviews, often systematic, have partially explored the topic of general practice-based pharmacists (GPBPs) internationally. Our aim was to further explore the employment/integration models of GPBPs and their actual activities and impact, concepts that have not been thoroughly investigated by previous reviews.

**Methods:**

Two databases were searched from inception to June 2021 for studies published in the English language. Results were independently screened by two reviewers to establish eligibility for inclusion. Original research studies, or protocols where results had not been released at the time of search, that reported on services provided by pharmacists with some sort of integration into general practices were included. The studies were analysed using narrative synthesis.

**Results:**

Searches identified 3206 studies in total, of which 75 met the inclusion criteria. The included studies were highly heterogeneous in terms of participants involved and methodologies employed. Integration of pharmacists into general practices has occurred in several countries, with funds originating from multiple sources. Several employment models for GPBPs were described – for example, part-time and full-time work and/or coverage of multiple or single practices. GPBP activities, with some exceptions, were comparable between different countries, with medication reviews being the most common task globally. GPBP impact was identified through both observational and/or interventional research methods, by pursuing a large variety of measures (e.g. activity volume, contact with patients, perceptions/experiences, and patient outcomes). Independent, quantifiable outcomes from GPBP activities were all positive but were of varying statistical significance.

**Conclusions:**

Our findings suggest that GPBP services can lead to positive, quantifiable outcomes, mainly in relation to medication use. This shows the usefulness of GPBP services. The findings of this review can help policy makers decide how best to implement and fund GPBP services, and how to identify and measure GPBP impact.

## Introduction

English general practices (known as ‘family practices’ in some countries) have been under significant workload pressures stemming from an ageing population with complicated health care needs.^1^ The recent pandemic further added to general practice workloads by generating extra tasks, including aiding the recovery of people physically or mentally affected by the pandemic, supporting patients on waiting lists for health care services, and contributing to the vaccination programme.^2^ In response to unmanageable workloads, general practitioners (GPs) are increasingly retiring early or switching to part-time employment patterns.^3,4^ As a result, there have been persistent shortfalls in the numbers of GPs.^3,5^

To tackle the workforce and workload pressures in general practices, there has been an extensive drive (begun in 2015) to integrate pharmacists into general practices. As part of a recent initiative to merge general practices in primary care networks, National Health Service England has endeavoured to fully sponsor the employment costs for hiring approximately 26,000 primary care staff by 2023/24 including additional general practice-based pharmacists (GPBPs).^6,7^ Primary care networks are collaborative structures linking primary care with hospital, social, and voluntary services, serving 30,000 to 50,000 people. It is anticipated that each of the approximately 1250 primary care networks will have about six pharmacists by 2023/24, thus elevating the population of GPBPs in England to about 7500.^8^ Similar to England, extensive attempts to integrate pharmacists into general practices have taken place in Scotland, Wales, and Northern Ireland.^9,10,11^

Formal integration of pharmacists into general practices is a relatively new concept in England. To demonstrate the value pharmacists add to the general practice setting and thereby justify their inclusion amongst the primary care team, National Health Service England has proposed a number of approaches over the years. These have included numerical and survey-based key performance indicators (i.e. quantifiable measures to track the progress of services or organisations in relation to process or outcomes), electronic activity codes to capture pharmacist activities, and non-pharmacist-specific measures relating to structured medication reviews, care in nursing homes, and cancer detection.^12,13,14^ Pursuing these impact identification plans, however, has been complicated by variations in pharmacist roles. There have been reports of GPBPs being resistant to the changes, dissatisfied with the available central measures (i.e. they regard the central measures as inappropriate for capturing GPBP impact), as well as inconsistencies in how pharmacist impact is ultimately identified between different practices.^15,16,17^

A number of systematic and non-systematic literature reviews have explored, to a certain extent, the topic of GPBPs around the globe. The reviews by Tan et al., Hazen et al., Anderson et al., Hayhoe et al., Ibrahim et al., Alshehri et al., and Khaira et al.^18,19,20,21,22,23,24^ collated outcomes from GPBP activities. The reviews by Benson et al.,^25^ as well as, to a lesser extent, those by Ibrahim et al. and Khaira et al.,^22,24^ investigated the types of activities carried out by GPBPs. One umbrella review^26^ also looked at existing systematic reviews, some of which reported on pharmacist services in primary care settings in general, rather than general practices specifically.

But none of these reviews described the different efforts of integrating pharmacists into general practices internationally or the methods used for identifying and measuring their impact. Moreover, although some of the reviews mentioned above considered the outcomes of GPBP activities, the described outcomes were not based solely on independent measures. Objective, independent measures might translate to different findings from those approaches that are subjective or self-reported for a given research topic/phenomenon.^27,28^ Further, some of the previous reviews related to GPBP services for specific conditions and/or certain patient populations only,^22,23^ or to GPBP services in very restricted geographical areas.^24^ In addition, the identified range of GPBP activities (in the review by Benson et al.^25^) also included student activity and potential roles, rather than focusing only on existing pharmacist services taking place in reality.

Thus, the overall aim of this rapid review was to shed more light on existing integration and employment models, actual roles, and impact of GPBPs. The specific questions this review set out to answer were as follows:

• What attempts have been made internationally to integrate pharmacists into general practice?
• What is the range of activities carried out by GPBPs?
• What methods have been used to identify the impact of GPBPs on practices and patients?
• What are the independent, quantifiable outcomes from GPBP activities discovered as part of impact identification methods?

## Methods

A rapid review is defined as ‘a form of knowledge synthesis that accelerates the process of conducting a traditional systematic review through streamlining or omitting a variety of methods to produce evidence in a resource-efficient manner’.^29(p80)^ We therefore selected this rapid review approach to produce timely results and inform the current implementation of GPBP services in the UK and overseas.

This study was carried out in accordance with the guidance for rapid reviews produced by the Cochrane Rapid Reviews Methods Group.^30^ This consists of 26 recommendations, in relation to designing, conducting, and writing up rapid reviews, as informed by a scoping review and extensive consultations with representatives from Cochrane entities. The objectives of and criteria for this review were developed collaboratively by GDK and NP, with the rest of the authors having input in reviewing decisions made and proof reading.

### Search strategy

To identify studies eligible for answering the objectives of this review, two databases (PubMed and Web of Science) were searched. The performed searches covered the period from inception of the databases until 11 June 2021. Search strategies were developed in collaboration with a subject librarian and involved the use of certain keywords, including ‘pharmacist’, ‘pharmacists’, ‘general practice’, and ‘family practice’. All search terms were combined by employing the Boolean operators ‘AND’ and ‘OR’ as appropriate. The precise search strategies are described in S1 in the online supplement. Reference lists of the included studies were also searched for additional relevant studies.

### Selection of studies

#### Study types

Any study reporting on original research, with a formal data collection method but regardless of the study design, was deemed eligible for inclusion in this review. We also included protocols for research studies in cases where results of the actual studies had not been released at the time of the search. All studies must have been published in peer-reviewed journals, written in the English language, and could have originated from any country across the globe. Letters to the editor, editorials, commentaries, experiences, special features, reports, research briefs, and systematic or any other type of literature review were excluded.

#### Participant types

There were no restrictions to the type of participants considered for this review. As such, to be included, studies could have involved any type of stakeholders in the implementation of GPBP services, such as patients, various health care professionals, and managerial or administration staff. There were no limits in relation to the kind of diseases patient participants experienced and/or GPBP participants dealt with.

#### Service types

We employed the definition of ‘integration’ by Shaw and Couzos. They described ‘integration’ as ‘any intervention that involved co-location of pharmacists within PHC [primary health care] settings and/or pharmacists who worked as part of multidisciplinary and/or interdisciplinary healthcare teams using a range of integrative processes’.^26(p404)^

Therefore, to be included in this review, studies must have involved the provision of pharmacist services in a community-based, general practice or family practice or primary care clinic setting. The actual tasks could have been undertaken either within the practice or remotely (e.g. patient homes or nursing homes or research sites). In any case, services should have been provided by pharmacists with some sort of integration into general practice but regardless of employment model.

We excluded studies describing the activity of student pharmacists in general practice or those examining the potential, rather than existing, activities and roles for GPBPs. We also excluded studies concerning pharmacist activity within community pharmacies, various types of clinics (e.g. memory clinics, occupational health clinics, and ambulatory care clinics – hospital-based services offering same day care to patients), outpatient settings that apart from primary care services also provided specialist care, and family medicine residency programmes. Finally, we excluded pharmacist-led educational projects and technological tools or manufacturer initiatives in general practice, as well as jointly delivered interventions (e.g. clinics carried out by GPBPs and nurses).

#### Outcome types

This review synthesised all methods that had been employed, by the time of our searches, to identify pharmacist impact in general practice. However, the specific outcomes discovered as part of these impact identification methods had to be based on independent, quantifiable measures. Outcomes of GPBP activities based on stakeholder opinions, including patient self-reporting or assumptions (e.g. cost savings computed to longer period of times than what was actually measured), were not presented in this review. The quantity of GPBP activities or interventions or patient encounters, including the duration of GPBP activities or encounters, was also not viewed as outcomes to present in this review.

#### Screening process

The title and abstract of all identified studies were screened by one reviewer (GDK) and studies that were outside the topic of GPBPs were excluded. All remaining, potentially relevant, studies were extracted to a reference management software. After removing duplicates, full texts were retrieved and independently read by two reviewers (GDK and NP) to establish whether inclusion criteria were met. GDK read all full-texts, whereas NP read a random sample of 50% of the full-texts. Both reviewers met regularly to discuss their findings and resolve any disagreements that arose. A list of studies fully satisfying the criteria was mutually agreed amongst the two reviewers.

#### Data extraction and synthesis

GDK independently extracted data from all included studies (see S2 in the online supplement). Details extracted included the study type and aim, the study population, information on the programmes of implementing GPBP services, the activities of GPBPs, the methods used for identifying GPBP impact, and key findings relevant to the objectives of this review. NP screened all extracted information, and its consistency and presentation were refined through discussions between GDK and NP.

No quality assessments for risk of biases were performed, as our purpose was to capture the broader picture in terms of integrating pharmacists into general practices globally rather than simply synthesising a narrow body of high-quality literature. In other words, as the primary purpose of this review was to synthesise the characteristics of GPBP models in relation to employment and impact identification, evaluating details about these models as being ‘present’ or ‘not present’ did not require any quality assessment techniques.

Data extracted from the included studies mainly consisted of descriptive information. The presence of descriptive elements, along with the fact that outcomes to be presented were heterogenic, meant that any meta-analyses could not be performed. We therefore selected a narrative synthesis approach to organise and present the extracted data. Narrative synthesis allowed us to ‘go beyond the act of simply describing and summarising the main features of included studies…enabling investigation of similarities and differences between studies, and exploration of relationships within the data’.^31(p201)^

## Results

### Selection of studies

**Figure 1.**
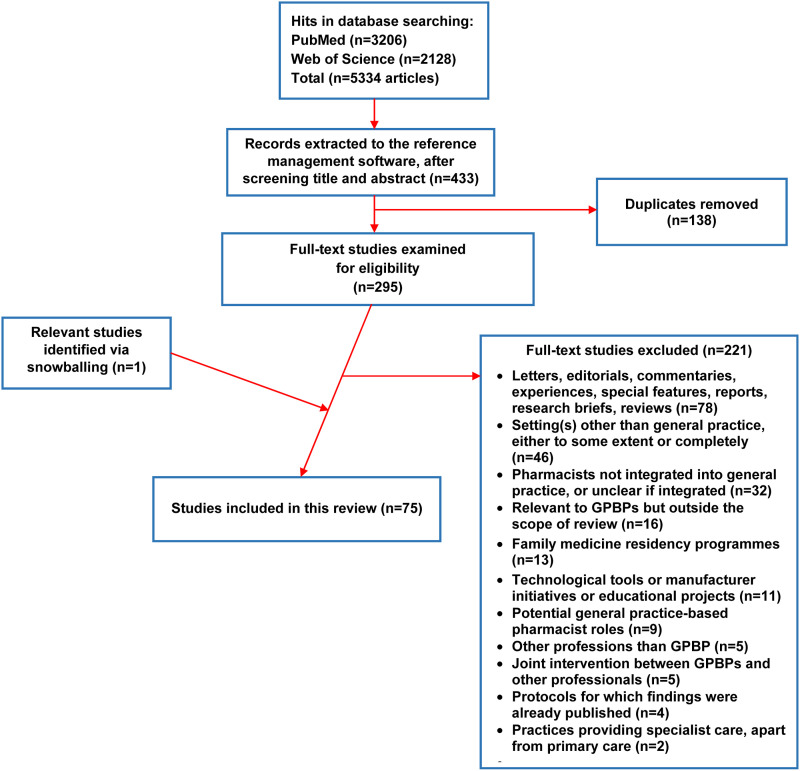
Flow diagram outlining the selection process of studies for the rapid review.

The original search resulted in 3206 studies in total. After screening titles and abstracts and removing duplicates, 295 studies were deemed potentially relevant. Full-texts were retrieved and read for all 295 studies. Seventy-five studies were regarded as eligible and included in this review. Figure 1 provides a detailed overview of the study selection process, including the precise reasons behind the exclusion of full-texts.

All included studies are summarised in S2 in the online supplement. The reference numbers in brackets below refer to the enumeration of the included studies as listed in S2 in the online supplement.

### Characteristics of included studies

Of the 75 included studies, 73 described original research (1–19, 21–67, and 69–75) and two were protocols for research studies for which findings had not been released by the time of the search (20 and 68). Twenty-five studies originated from the UK (1, 8, 9, 11, 18, 33, 38, 42–45, 47–50, 52–54, 56, 60, 62, 63, 67, 69, and 75), 19 from the US (10, 14–17, 19, 29–32, 34, 39, 41, 51, 55, 59, 61, 64, and 73), 13 from Australia (2, 4–6, 21–23, 26, 46, 68, and 70–72), nine from Canada (3, 7, 24, 25, 27, 28, 57, 58, and 74), four from the Netherlands (36, 37, 65, and 66), three from the Republic of Ireland (13, 20, and 40), and two from New Zealand (12 and 35). Studies were highly heterogeneous concerning the type of participants involved (e.g. pharmacists, patients with varied conditions, GPs and other practice staff, scheme commissioners, and training leads) as well as the designs and methodologies described (e.g. observational research, controlled trials, pilot studies, surveys, qualitative studies, and mixed-methods research). Around 80% of the included studies were published in the last 10 years, of which 75% were published from 2018 onwards.

### Integrating pharmacists into general practices internationally

Efforts to integrate pharmacists into general practices have taken place in several countries since the late 1990s. GPBP services have been implemented, to a large degree, through short-lived programmes lasting from 4 months to a few years (10, 13, 15, 19–24, 29, 30, 36, 37, 39, 40, 46–49, 57, 58, 60, 65, 66, 68, and 70–73). The UK is the only country with established nationwide programmes so far. In other countries, efforts to integrate pharmacists were restricted to specific geographical locations and/or were of a small scale in terms of number of general practices and pharmacists involved (3–7, 10, 12–17, 19–32, 34–37, 39–41, 46, 51, 55, 57–59, 61, 64–66, 68, and 70–74).

A number of funding sources for GPBP services were reported (2–6, 8, 11–13, 21–24, 28, 32, 34, 35, 38–50, 54, 57–62, and 67–72). Funds originated from general practices themselves (including from billing patients), local primary care or other clinical structures, universities (in cases where there was some affiliation between practices and local universities), specific research teams (where grants were obtained to integrate pharmacists into general practice for a certain time period and study their impact), and governments. It is therefore apparent that a common funding model for GPBPs is absent, either inside the same country or globally. However, in general, programmes led and funded by governments appear to lead to larger numbers of GPBP posts, with the highest numbers being in Canada and the UK.

A large variety in the models of employment and integration also exists. In most studies, pharmacists were reported to work part-time in their general practice-based role spending the rest of their working time on parallel affiliations such as community pharmacy, hospitals, specialist services, and other clinical and non-clinical bodies, including academia (2–6, 10, 11, 13, 15, 19, 20–24, 28–31, 38–41, 46, 48, 49, 51, 57–59, 67, 68, and 70–72). Full-time employment in general practice-based roles was less common and was present in the ‘Pharmacotherapy Optimisation through Integration of a Non-dispensing pharmacist in a primary care Team’ programme in Netherlands, in one local effort in the US, in the Canadian province of Ontario, in New Zealand, and in the UK (but only after the large governmental schemes commenced in 2015) (1, 12, 27, 35–37, and 64–66).

In some cases, pharmacists were directly employed by practices (13, 20–23, 28, 36, 37, 40, 49, 65, 66, 68, and 70–72), whereas elsewhere pharmacists were officially hired by other structures (e.g. private companies, primary care structures, clinical bodies, specialist services, and universities) and integrated into general practices (3, 11, 12, 24, 25, 27, 35, 38, 47, 57–59, and 62). Coverage of multiple practices (i.e. one pharmacist serving a number of general practices) was a common phenomenon (1, 4–6, 10, 11, 13, 16, 17, 20, 27, 35, 40, 47, 48, 60, 63, and 67), but instances of pharmacists being located in one practice exclusively were also described (1, 3, 7, 19, 26–28, 34, 35, 40, 41, 55, 59, 61, 73, and 74). General practice-based pharmacists were either directly accessible to patients, as a choice equal to the GP and other health care professionals in general practice, or patients were invited to a pharmacist consultation (either directly by GPBPs or through referrals by other professionals) if they met certain criteria.

### Activities carried out by GPBPs

Different, and often conflicting, terminologies used amongst different studies and countries complicated the process of synthesising the GPBP activities internationally. An attempt has been made to organise and present activities as per the definitions provided below and in Table 1. A wide range of GPBP activities were described in the included studies (see Table 1). Our aim was not to quantify the range of services or comment on whether certain GPBP tasks were more frequently undertaken than others; therefore, activities presented in Table 1 are simply listed in alphabetical order. However, when examining the data extraction table (see S2 in the online supplement), it was apparent that medication reviews were commonly reported for GPBPs (across the globe) in most of the included studies (1–6, 8–13, 15–24, 26–32, 34–43, 46–49, 51, 53, 56–70, 72, 73, and 75). A medication review is the process of maximising the effectiveness of a patient’s pharmacotherapy by obtaining medication histories and relevant information (from the patient, their family, and/or the patient notes) and initiating, stopping, or amending medications or devices, as per the patient needs (e.g. contra-indications, interactions, and other cautions). Both face-to-face (within general practices or patient homes or residential aged-care facilities) and note-based medication reviews (without the physical presence of the patient) were described in the included studies. Medication reviews by GPBPs involved either all medications patients were taking or medications for certain conditions only.

**Table 1.** Common GPBP activities, as presented in the included studies (listed in alphabetical order).

GPBP activities	S2 references in online supplement
Acute care	(8, 11, and 62)
Adherence checking^a^	(2, 30, 69, 70, and 72)
Care coordination by liaising with various primary and secondary care settings	(2, 8, 12, 13, 40, 46, 49, 50, and 67)
Development of formal care plans for patients	(3, 9, 15, 17, 21, 23, 25, 29, 30, 36, 37, 39, 51, 53, 55, 57, 58, 61, 64, 65, 66, and 69)
Direct prescribing duties	(8, 9, 11, 31, 38, 42, 43, 50, 53, 62, and 63)
Lifestyle advice, for example, on diet, weight management, smoking cessation, and sleep hygiene	(2, 4–6, 15, 22, 23, 30, 32, 39, 46, 47, 59, 69, 70, and 72)
Long-term condition and/or polypharmacy management	(2, 3, 8, 11, 20, 21, 25, 28, 35, 55, 57, 58, 62, and 63)
Management/screening repeat prescriptions	(3, 8, 13, 20, 40–43, and 60)
Medication reviews	(1–6, 8–13, 15–24, 26–32, 34–43, 46–49, 51, 53, 56–70, 72, 73, and 75)
Medicine reconciliations^b^	(1, 2, 8, 11, 12, 16, 20, 27, 35–37, 42, 43, 46, 49–51, 59, and 62–66)
Participating in and/or leading multidisciplinary team meetings	(11, 13, 42, and 43)
Patient counselling on conditions, medications, and devices	(2, 4–7, 10, 14, 15, 17, 21–23, 26, 27, 29, 30, 32, 39, 41, 46, 51, 55, 59, 61, 64, 68–72, and 74)
Patient following up and/or monitoring of their condition or medications, including direct ordering of tests	(3, 8, 10, 11, 15, 20, 24, 30–32, 34, 39, 46, 51, 55, 57–59, 61, 62, and 69)
Physical assessments	(8, 42, 43, 61, and 62)
Practice staff education, including educational sessions, support on a one-to-one basis, and student supervision	(2, 8, 12–15, 17, 20, 22–30, 36, 37, 39–43, 50, 57, 58, 65, 66, 68, and 71)
Quality management tasks, such as prescribing audits (e.g. on high-risk drugs), medication use projects, incentive programmes, and synthesis of policies, protocols, bulletins etc	(2, 8, 11–13, 17, 20, 22, 24, 25, 36, 37, 42–43, 60, 62, 63, 67, 68, and 71)
Telephone consultations with patients, including for triage purposes	(1, 7, 8, 15, 17, 20, 28–30, 39, 42, 43, and 62)

^a^Adherence checking relates to the process of establishing whether the patient uses medications or devices as intended (e.g. checking the patient’s inhaler technique).

^b^Medicine reconciliation is the process of updating medication lists/records so to reflect what patients are actually taking/should be taking, by also taking into consideration various types of correspondence during transfer of care (e.g. hospital discharge letters or outpatient letters).

In general, reported GPBP activities were quite comparable between different countries. One exception was smoking cessation services, which were only specifically mentioned in Australian studies (2 and 46). Other exceptions were the management of high-risk drugs and engagement with incentive programmes, which were only reported in the UK (8, 11, 42, 43, and 62). Examples of incentive programmes included the Quality and Outcomes Framework (a programme for English, Welsh, and Northern Irish general practices that incentivises clinical excellence) and the Quality, Innovation, Productivity and Prevention scheme (a combination of programmes in England to ensure that money is spent in a way that maximises the quality of care and benefits for patients).

Provision of acute care, physical assessments, and direct ordering of laboratory/clinical tests were also reported in the UK-based studies, as well as at some local schemes in the US (8, 11, 31, 32, 42, 43, 59, 61, and 62). Although the management and/or screening of repeat prescriptions were amongst GPBP tasks in several countries (3, 8, 13, 20, 40–43, and 60), direct prescribing and authorisation of repeat prescriptions were reported only in the UK and in the US (at a much smaller extent) (8, 9, 11, 31, 38, 42, 43, 50, 53, 62, and 63). Overall, UK GPBPs appeared to work more independently from GPs than other countries, where pharmacist input ended with a number of recommendations awaiting approval and implementation by GPs.

### Methods used for identifying GPBP impact

To capture GPBP impact, the studies used both observational and interventional research designs, sometimes in combination. Observational studies were either descriptive or exploratory in nature, and pursued a plethora of survey, qualitative, or mixed-methods approaches. Interventional studies included various types of controlled trials (often randomised) and/or before–after procedures. Research efforts were often carried out in the form of pilot studies. Table 2 summarises the specific impact identification methods commonly encountered in the studies.

**Table 2.** Common methods used for identifying GPBP impact, as described in the included studies (listed in alphabetical order).

Methods	S2 references in online supplement
Calculation of acceptance rates of GPBP recommendations	(4, 5, 13, 16, 37, 64, and 73)
Comparisons between GPBP-led and usual care, as part of controlled trials and/or before–after approaches, based on	(9, 13–16, 19–21, 26, 29, 30, 32, 34, 38, 41, 47, 50, 51, 53, 55, 59, 60, 64–66, 69, 71, 72, 74, and 75)
•Treatment and prescribing appropriateness (various measures)	
•Use of medications and associated problems (various measures)	
•Status and control of long-term conditions (various measures)	
•General health and satisfaction, including quality of life and anxiety	
•Use of primary and hospital care and associated costs (various measures)	
Extrapolations about savings in GP time and cost-savings in practices, based on the volume of GPBP input in general practices	(11, 13, 46, and 73)
Quantification of	(3–6, 10, 11, 13, 17, 20, 21, 23, 26, 34, 37–39, 41–43, 46, 49, 51, 64, and 73)
•Daily work elements (e.g. numbers of GPBP activities and recommendations)	
•Contact with patients, practice staff, and community pharmacies	
•Medication-related problems^a^ identified and/or resolved	
Use of questionnaires and/or interviews and/or focus groups with various stakeholders to elicit	(6, 7, 20–22, 24, 27, 31, 36, 44, 45, 48–50, 52, 56, 57, 62, 63, and 68–70)
•Experiences of and/or satisfaction with GPBP services	
•Perceived impact on various aspects of patient care and work of professionals including on the dynamics and collaboration within multidisciplinary teams	

^a^Medication-related problems are circumstances or events in relation to pharmacotherapy that possibly reduce the chances for an ideal outcome for the patient, such as adverse drug reactions, interactions, inappropriate doses or storage conditions, and untreated indications.

### Independent, quantifiable outcomes from GPBP activities

Independently measured, quantifiable outcomes were reported in only 32 of the included studies (4, 5, 13–17, 19, 26, 29–32, 34, 37, 38, 41, 47, 50, 51, 55, 59–61, 64–66, 69, and 71–75), of which half originated from the US. Characteristic examples of independent, quantifiable outcomes of the presence of pharmacists in general practice are presented in Table 3.

**Table 3.** Independent, quantifiable outcomes from GPBP activities, as reported in the included studies (listed in alphabetical order).

Outcomes	S2 references in online supplement
Cost-reductions for general practices, for example, due to more efficient medication prescribing, medication use, and use of services	(26, 60, 69, 73, and 75)
Enhanced use of medications at practices, expressed as	(14, 16, 19, 30, 32, 34, 38, 47, 55, 59, 69, 71, and 75)
•Higher percentage of treatments in accordance with clinical guidelines	
•Reductions in overall or certain medications	
•Increased overall prescribing rates for and/or use of agents necessary in certain conditions	
Fewer hospitalisations and Accident and Emergency attendances, as well as less use of general practice services	(29, 51, 65, and 69)
Improvements in the status of certain conditions, usually expressed as changes in clinical values such as International Normalised Ratio, blood pressure, glycated haemoglobin, and cholesterol	(15, 19, 34, 41, 47, 59, 61, and 74)
Patients experiencing fewer medication-related problems due to increased identification and resolution of medication-related problems, including high percentages of GPBP recommendations being implemented by general practitioners	(4, 5, 13, 16, 17, 34, 37, 64, 69, 72, and 73)
Time-saving for GPs	(50)

The significance of the measured differences fluctuated, with statistically significant changes in some cases (14–16, 29, 30, 32, 34, 51, 59–61, 69, 71, 72, and 74) and non-statistically significant differences and/or differences of unclear statistical significance elsewhere (19, 26, 38, 41, 47, 50, 51, 55, 64, 65, 69, and 75). Some studies found that GPBP introduction made no difference in terms of the achievement of clinical goals for long-term conditions (15, 16, and 34), use of primary and secondary care (51, 64, 69, and 75), quality of GP prescribing (66), and costs (65). However, no studies described any negative outcomes for patients and practices following pharmacist integration. Overall, we can say then that the outcomes of GPBP activities were largely positive.

## Discussion

Our rapid review found that the integration of pharmacists into general practices has occurred in seven countries, through a variety of employment and funding models. GPBPs engage in many activities and roles that, with some exceptions, are comparable across different countries. A wide spectrum of methods was used to identify GPBP impact, as part of observational and/or interventional research designs. Independently measured, quantifiable outcomes from GPBP activities were reported in less than a half of the included studies. All of these outcomes were positive but were of varying statistical significance.

There was a wide variety of research methodologies employed. This heterogeneity is partly a function of the inclusion criteria for this rapid review (which were rather broad) but could also be due to different stages in the development and implementation of GPBP services across different countries. When assessing health care interventions, formative methodologies (focusing on strengths and limitations of implementation strategies) tend to be used in early days of implementation, whereas more summative approaches (focusing on outcomes) are usually preferred in later stages of assessment.^32^ As the UK has had pharmacists in general practice the longest and in the highest numbers, it is no surprise that a great number of the included literature (a third of the studies) came from the UK.

There was a large variation in models of employment and integration for GPBPs, and in health care systems and/or funding between countries. No GPBP presence was found in certain regions of the developed world – for example, the Middle East and Eastern parts of Europe. Based on the included studies, it was not possible to determine what the best model for integration of GPBPs was. There was also no variation in outcomes arising from differences in GPBP models (e.g. full- or part-time posts or employment by different bodies linking to any more or less favourable outcomes). The only common theme in the literature was that governmental schemes translated to larger financial investments, more GPBP posts, greater longevity of the role, and a larger extent of full-time employment in general practice for GPBPs. Pharmacists’ presence full-time in single general practices was previously found beneficial for patients in terms of accessibility to services^33,34,35^ and might be worth considering when GPBP employment is desired.

As full integration leads to positive, patient-related outcomes and improvements in patient-centred, pharmacy services,^19^ several strategies have been considered internationally to support the integration of GPBPs into the primary care team. Examples include ensuring certain GPBP attributes (e.g. ability to build relationships with staff and patients, non-judgemental attitude, resilience, and clinical skills) are practised when working in general practice, employing GPBPs in practices that had previously worked with a pharmacist and thereby understood pharmacist capabilities, having a system enabling patients to self-refer to the pharmacist, and drawing on GPBP experience of the local community, through either previous local work or cultural orientation programmes and/or interactions with local community pharmacists.^36^ Successful GPBP integration was often found to depend on the availability of shared information systems (i.e. GPBPs working on same clinical record systems with the rest of the practice team),^19^ as well as on GPBPs bringing medication-related expertise into general practices and reconciling interprofessional tensions with other members of the practice team (caused by overlapping tasks) (36).

GPBP activities were wide-ranging and generally confirmed roles described in previous reviews.^22,24,25^ The fact that medication reviews were the most common task for GPBPs is unsurprising as it has been reiterated (by GPs and pharmacists themselves) that the focus of GPBPs should/could be the performance of complex, clinical medication reviews in line with pharmacist expertise and training.^37,38,39,40,41^ The larger degree of independence of UK GPBPs in their work might be attributed to their being able to independently prescribe (if qualified to do so), which allows pharmacists to directly make patient- and medication-related decisions.^42^ In contrast, in other countries pharmacists are not able to prescribe or can only do so under very specific conditions.

Since the advent of the recent pandemic, face-to-face medication reviews with patients in the general practice setting have largely been conducted virtually.^43^ This was not commonly encountered in the included studies. As a result, there is a chance that virtual GPBP services might diverge from services provided in person in terms of how their impact needs to be identified, also bearing in mind the intricacies of studying the use of technology in health care,^44^ and of determining outcomes. Associating specific activities with GPBP impact is an area of ambiguity (e.g. what GPBP actions exactly are responsible for certain positive outcomes?).

There has not been a common method for identifying GPBP impact, either within the same nation or across countries. A large number of measures have been employed to capture GPBP impact globally, such as activity volume, contact with patients, perceptions/experiences, and patient outcomes. An e-Delphi study found that funding acquisition for general practices was the most likely area for GPBPs to show a difference (42). Most of the included studies in this review, however, reported quantitative differences in relation to the use of medications (4, 5, 13, 14, 16, 17, 19, 30, 32, 34, 37, 38, 47, 55, 59, 64, 69, 71–73, and 75) rather than financial benefits, following pharmacist presence in general practice.

Quantitative improvements in clinical indicators (e.g. blood pressure, International Normalised Ratio, cholesterol, and glycated haemoglobin) were also described in the included studies (15, 19, 34, 41, 47, 59, 61, and 74). However, these findings only constitute a snapshot of these clinical indicators. Differences in clinical indicators do not necessarily translate to impact over respective long-term conditions, especially since GPBP integration and associated studies often lasted for short time periods and full control of long-term conditions might require a longer time period. It is also unclear if GPBPs would have more influence on any particular clinical indicator as listed above.

Some of the studies reporting on quantitative outcomes (mainly those accounting for use of hospital services and clinical indicators) found no differences and/or were inconclusive about the benefits of pharmacist integration into general practices (13, 15, 16, 19, 29, 30, 34, 38, 51, 64–66, 69, and 75). The difficulty of GPBPs to make a positive difference in the use of primary and secondary services, due to their dependence on various factors outside pharmacist control, has been echoed elsewhere (43). Where changes in relation to quantitative outcomes from GPBP activities were reported, only some were statistically significant. But even where changes were not significantly significant, GPBPs are still of value for general practices to assist GPs who are under increasing workload pressures in many countries of the Western world.^45^ Indeed, GPs in several countries have largely been satisfied with GPBPs and recognised GPBPs’ role in reducing GP workload and increasing GP confidence concerning medication-related matters.^46,47,48^

With regards to the UK, some studies accounted for some of the formal measures developed to capture GPBP impact (e.g. volume of medication reviews, Quality and Outcomes Framework-related targets for long-term conditions, and Accident and Emergency attendances) (11, 47, 49, and 69). No studies, however, explored GPBP impact on nursing home care and detection of cancer, areas contained in latest measures for GPBPs at a national level.^13,14^ Although the main goal of recent UK schemes was to alleviate pressures on GPs, just one study set out to independently measure actual reductions in GP workload (50). Overall, only six UK studies reported independently measured, quantifiable outcomes (38, 47, 50, 60, 69, and 75). Despite limited exploration of quantitative outcomes, there have been continuing investments to maintain and expand GPBP presence and roles in the UK, as there are now many positive indications – if not certainty – about the usefulness of GPBPs.

It is therefore questionable whether impact identification processes are still necessary for UK GPBPs and, more generally, wherever large-scale efforts of integrating GPBPs have taken place and/or GPBP services have existed for quite a while. Identifying impact is not a straightforward procedure, as impact is multidimensional, can have more than one cause, and may have a subjective element to it, making it hard to measure numerically.^49^

This review did not uncover what the easiest and/or best way to measure GPBP impact is. In the studies it was often reported that randomised controlled trials, for example, are the gold standard for impact research, since randomisation minimises biases by ensuring attribution of measured differences to the intervention of interest.^50^ Although such trials might allow for certain outcomes to be followed over time, that may mean the findings are not of great relevance by the time they are published due to the rapidly changing landscape in health care.^51^ Randomised controlled trials are also characterised by inherent ‘experimental’ elements that do not necessarily reflect real-world situations.^52^ For example, it is difficult to set up an experiment to measure GPBP impact when GPBP work is multidimensional in nature and integrated care is collaboratively provided by GPBPs and general practice teams. As such, there are claims that non-randomised controlled trial quasi-experimental, before–after studies might be more successful in identifying real-world impacts.^51,52^ Perhaps an easier way to assess the usefulness of GPBPs, as well as any effect of role and/or skillset variation, could be through appraisal processes (i.e. sessions between line manager and employee to evaluate employee’s performance against mutually agreed objectives),^53,54^ which do not require research expertise and have historically been performed for all clinical staff in general practices.

## Limitations

There are three main limitations to the current study. First, because it was not a full-systematic review there might have been additional literature on the topic of GPBPs, indexed in other databases, that was not captured. The fact only studies in English were considered means information about GPBPs published in other languages, and hence about other countries, may have been missed. In addition, it might have been that further details about GPBP models and activities were contained in grey literature reports (e.g. policy documents), which were not included in this review.

Second, the included protocol papers provided detailed information on what the respective study teams aimed to do in relation to employing and identifying GPBP impact. However, the fact that studies were not completed meant that we do not know what the impact of these GPBPs actually was.

Third, the risk of biases in the findings of the review could not be completely eliminated due to the fact no quality assessments were performed and that narrative synthesis is inherently subjective. This means different research teams might reach slightly different conclusions from the same literature. In addition, the fact that clinical endpoints and outcome measures reported in the included studies were not critically appraised (via quality assessment tools) might mean that the trustworthiness of the findings of this review pertaining to measurable GPBP impact might be questioned. However, other reviews that did engage with quality assessments^18,19,21,22,23^ reported similar (positive) findings to this rapid review in relation to GPBP impact on patients and health care professionals.

## Conclusions

Our findings validate the usefulness of GPBP services for patients and practices, by demonstrating positive quantifiable outcomes, especially in relation to medication use. Moreover, this review shows that there are many GPBP models and ways of identifying GPBP impact. Government funding is worth considering when large-scale and long-term integration of pharmacists into general practices is desired.

For countries that want to develop GPBP services, this review provides ideas for how to do this and what evidence might be required to justify pharmacist inclusion in general practice. According to the findings of this review, it is more likely for pharmacist integration into general practices to impact upon medication use rather than use of health care services (e.g. Accident and Emergency attendances, hospitalisations, and consultations in general practice).

Future research efforts should focus on measuring quantifiable outcomes from GPBP activities for which there is ambiguity and/or no conclusive evidence. These include hospital admissions and/or Accident and Emergency attendances, clinical indicators for long-term conditions, and cost-savings. Clinical indicators need to be followed up in the long term to establish whether the GPBP services have a long-term impact. Prospective studies should also attempt to quantify the precise time-savings for GPs, post-implementation of GPBP services, as well as establish any statistically significant associations between GPBP models and improvements in quantifiable outcomes. Such research would identify the most beneficial GPBP models for patients and practices.

## Supplemental Material

Supplemental Material - Integration and impact of pharmacists in general practice internationally: A rapid review Click here for additional data file. Supplemental Material for Integration and impact of pharmacists in general practice internationally: A rapid review by Georgios Dimitrios Karampatakis, Nilesh Patel, Graham Stretch and Kath Ryan in Journal of Health Services Research & Policy

## References

[1] Baird B Charles A Honeyman M , et al. Understanding pressures in general practice. London: The King’s Fund, 2016, https://www.kingsfund.org.uk/sites/default/files/field/field_publication_file/Understanding-GP-pressures-Kings-Fund-May-2016.pdf (accessed 15 May 2019).

[2] Bostock N . GP practices “at breaking point” after 20% surge in appointments last month. GPonline https://www.gponline.com/gp-practices-at-breaking-point-20-surge-appointments-last-month/article/1714359 (2021), (accessed 15 June 2021).

[3] British Medical Association . Pressures in general practice, 2021, https://www.bma.org.uk/advice-and-support/nhs-delivery-and-workforce/pressures/pressures-in-general-practice (accessed 15 June 2021).

[4] Bostock N . More than one in three GPs plan early retirement as pandemic and workload take toll. GPonline, 2021, https://www.gponline.com/one-three-gps-plan-early-retirement-pandemic-workload-toll/article/1714669 (accessed 15 June 2021).

[5] Campbell D . Shortage of GPs will never end, health experts say. London, UK: The Guardian, 2019, https://www.theguardian.com/society/2019/mar/21/shortage-of-gps-will-never-end-health-experts-say (accessed 17 July 2020).

[6] NHS England British Medical Association . Update to the GP contract agreement 2020/21 - 2023/24, 2020, https://www.england.nhs.uk/wp-content/uploads/2020/03/update-to-the-gp-contract-agreement-v2-updated.pdf (accessed 7 May 2020).

[7] NHS England The NHS long term plan, 2019, https://www.longtermplan.nhs.uk/wp-content/uploads/2019/01/nhs-long-term-plan.pdf (accessed 17 May 2019).

[8] Andalo D . Number of clinical pharmacists expected to work in PCNs rises to 7,500 by 2023/2024 Pharm J 2019, https://www.pharmaceutical-journal.com/news-and-analysis/news/number-of-clinical-pharmacists-expected-to-work-in-pcns-rises-to-7500-by-2023/2024/20207066.article (accessed 19 September 2019).

[9] Parr RM . Enhancing pharmacy services across NHS Scotland. Pharm J 2018, https://www.pharmaceutical-journal.com/opinion/comment/enhancing-pharmacy-services-across-nhs-scotland/20204538.article (accessed 14 July 2018).

[10] NHS Wales . Clinical pharmacists in GP practices, 2016, http://www.wales.nhs.uk/news/40188 (accessed 28 March 2018).

[11] Department of Health . £26.76 million investment in GP services announced, 2019, https://www.health-ni.gov.uk/news/ps2676-million-investment-gp-services-announced (accessed 29 August 2020).

[12] NHS England Health Education England . Clinical pharmacists in general practice pilot, 2015, https://www.england.nhs.uk/commissioning/wp-content/uploads/sites/12/2015/07/clinical-pharmacists-gp-pilot.pdf (accessed 13 October 2016).

[13] NHS England . Network contract directed enhanced service: contract specification 2020/21-PCN requirements and entitlements, 2020, https://www.england.nhs.uk/wp-content/uploads/2020/03/network-contract-des-specification-pcn-requirements-entitlements-2020-21.pdf (accessed 19 August 2020).

[14] NHS England . Network contract direct enhanced service-draft outline service specifications, 2019, https://www.engage.england.nhs.uk/survey/primary-care-networks-service-specifications/supporting_documents/Draft_PCN_Service_Specifications_December_2019.pdf (accessed 19 August 2020).

[15] Hampson N Ruane S . The value of pharmacists in general practice: perspectives of general practitioners-an exploratory interview study. Int J Clin Pharm 2019; 41: 496–503. 30864082 10.1007/s11096-019-00795-6

[16] Mann C Anderson C Avery A , et al. Clinical pharmacists in general practice: pilot scheme evaluation. Nottingham, UK: University of Nottingham, 2018, https://www.nottingham.ac.uk/pharmacy/documents/generalpracticeyearfwdrev/clinical-pharmacists-in-general-practice-pilot-scheme-full-report.pdf (accessed 24 August 2018).

[17] Karampatakis GD Ryan K Patel N , et al. How do pharmacists in English general practices identify their impact? An exploratory qualitative study of measurement problems. BioMed Central 2019; 19: 34. 10.1186/s12913-018-3842-y PMC6332895 30642315

[18] Tan ECK Stewart K Elliott RA , et al. Pharmacist services provided in general practice clinics: a systematic review and meta-analysis. Res Social Adm Pharm 2014; 10: 608–622. 24161491 10.1016/j.sapharm.2013.08.006

[19] Hazen ACM de Bont AA Boelman L , et al. The degree of integration of non-dispensing pharmacists in primary care practice and the impact on health outcomes: a systematic review. Res Social Adm Pharm 2018; 14: 228–240. 28506574 10.1016/j.sapharm.2017.04.014

[20] Anderson C Zhan K Boyd M , et al. The role of pharmacists in general practice: A realist review. Res Social Adm Pharm 2019; 15: 338–345. 29907317 10.1016/j.sapharm.2018.06.001

[21] Hayhoe B Cespedes JA Foley K , et al. Impact of integrating pharmacists into primary care teams on health systems indicators: a systematic review. Br J Gen Pract 2019; 69: e665–e674. 31455642 10.3399/bjgp19X705461 PMC6713515

[22] Hasan Ibrahim AS Barry HE Hughes CM . A systematic review of general practice-based pharmacists’ services to optimize medicines management in older people with multimorbidity and polypharmacy. Fam Pract 2021; 38: 509–523. 33506870 10.1093/fampra/cmaa146

[23] Alshehri AA Jalal Z Cheema E , et al. Impact of the pharmacist‐led intervention on the control of medical cardiovascular risk factors for the primary prevention of cardiovascular disease in general practice: a systematic review and meta‐analysis of randomised controlled trials. Br J Clin Pharmacol 2020; 86: 29–38. 31777082 10.1111/bcp.14164 PMC6983518

[24] Khaira M Mathers A Benny Gerard N , et al. The Evolving Role and Impact of Integrating Pharmacists into Primary Care Teams: Experience from Ontario, Canada. Pharmacy 2020; 8: 234. 33297509 10.3390/pharmacy8040234 PMC7768418

[25] Benson H Lucas C Benrimoj SI , et al. The development of a role description and competency map for pharmacists in an interprofessional care setting. Int J Clin Pharm 2019; 41: 391–407. 30879217 10.1007/s11096-019-00808-4

[26] Shaw C Couzos S . Integration of non-dispensing pharmacists into primary healthcare services: an umbrella review and narrative synthesis of the effect on patient outcomes. Aust J Gen Pract 2021; 50: 403–408. 34059845 10.31128/AJGP-08-20-5565

[27] Oates GR Stepanikova I Rowe SM , et al. Objective versus self-reported adherence to airway clearance therapy in cystic fibrosis. Respi Care 2019; 64: 176–181. 10.4187/respcare.06436 PMC6818680 30538158

[28] Prince SA Adamo KB Hamel ME , et al. A comparison of direct versus self-report measures for assessing physical activity in adults: a systematic review. Int J Behav Nutr Phys Act 2008; 5: 56. 18990237 10.1186/1479-5868-5-56 PMC2588639

[29] Hamel C Michaud A Thuku M , et al. Defining rapid reviews: a systematic scoping review and thematic analysis of definitions and defining characteristics of rapid reviews. J Clin Epidemiol 2020; 129: 74–85. 33038541 10.1016/j.jclinepi.2020.09.041

[30] Garritty C Gartlehner G Nussbaumer-Streit B , et al. Cochrane rapid reviews methods Group offers evidence-informed guidance to conduct rapid reviews. Journal of Clinical Epidemiology 2020; 130: 13–22. 33068715 10.1016/j.jclinepi.2020.10.007 PMC7557165

[31] Lisy K Porritt K . Narrative Synthesis. Int J Evid Based Health 2016; 14: 201.

[32] World Health Organization . Monitoring and evaluating digital health interventions: a practical guide to conducting research and assessment, 2016, https://saluddigital.com/wp-content/uploads/2019/06/WHO.-Monitoring-and-Evaluating-Digital-Health-Interventions.pdf (accessed 8 February 2022).

[33] Claire M Claire A Matthew B . The role of clinical pharmacists in general practice in England: Impact, perspectives, barriers and facilitators. Res Social Adm Pharm 2022; 18: 3432–3437. Epub ahead of print 29 October 2021. DOI: 10.1016/j.sapharm.2021.10.006 34802958

[34] Tan ECK Stewart K Elliott RA , et al. Stakeholder experiences with general practice pharmacist services: a qualitative study. BMJ Open 2013; 3: e003214. 10.1136/bmjopen-2013-003214 PMC3773653 24030867

[35] Savickas V Foreman E Ladva A , et al. Pharmacy services and role development in UK general practice: a cross-sectional survey. Int J Pharm Pract 2021; 29: 37–44. 32627272 10.1111/ijpp.12653

[36] Drovandi A Smith D Preston R , et al. Enablers and barriers to non-dispensing pharmacist integration into the primary health care teams of Aboriginal community-controlled health services. Res Social Adm Pharm 2022; 18: 3766–3774. 35581127 10.1016/j.sapharm.2022.05.002

[37] Ackermann E Douglas Williams I Freeman C . Pharmacists in general practice--a proposed role in the multidisciplinary team. Aust Fam Physician 2010; 39: 163–164. 20369121

[38] Stone MC Williams HC . Clinical pharmacists in general practice: value for patients and the practice of a new role.Br J Gen Pract 2015; 65: 262–263. 25918328 10.3399/bjgp15X685033 PMC4408499

[39] Turner JP Bell JS . Implementation of pharmacist-led medication reviews in general practice. Int J Clin Pharm 2013; 35: 3–4. 23129419 10.1007/s11096-012-9721-4

[40] Williams S Hayes J Brad L . Clinical pharmacists in general practice: a necessity not a luxury? Br J of Gen Pract 2018; 68: 85–85. 29371307 10.3399/bjgp18X694697 PMC5774951

[41] Tan ECK Stewart K Elliott RA , et al. Integration of pharmacists into general practice clinics in Australia: the views of general practitioners and pharmacists. Int J Pharm Pract 2014; 22: 28–37. 23750666 10.1111/ijpp.12047

[42] Cope LC Abuzour AS Tully MP . Nonmedical prescribing: where are we now? Ther Adv Drug Saf. 2016; 7: 165–172. 27493720 10.1177/2042098616646726 PMC4959632

[43] Baird B. How has general practice responded to the Covid-19 (coronavirus) outbreak? The King’s Fund, 2020, https://www.kingsfund.org.uk/blog/2020/04/covid-19-general-practice (accessed 9 July 2020).

[44] Guo C Ashrafian H Ghafur S , et al. Challenges for the evaluation of digital health solutions-A call for innovative evidence generation approaches. Npj Digit Med 2020; 3: 110. 32904379 10.1038/s41746-020-00314-2 PMC7453198

[45] Schäfer WLA van den Berg MJ Groenewegen PP . The association between the workload of general practitioners and patient experiences with care: results of a cross-sectional study in 33 countries. Hum Resour Health 2020; 18: 76. 33066776 10.1186/s12960-020-00520-9 PMC7565810

[46] Hasan Ibrahim AS Barry HE Hughes CM . General practitioners’ experiences with, views of, and attitudes towards, general practice-based pharmacists: a cross-sectional survey. BMC Primary Care 2022; 23: 6. 35172734 10.1186/s12875-021-01607-5 PMC8759266

[47] Hurley E Gleeson LL Byrne S , et al. General practitioners’ views of pharmacist services in general practice: a qualitative evidence synthesis. Fam Pract 2022; 39: 735–746. Epub ahead of print 26 September 2021. DOI: 10.1093/fampra/cmab114 34564715 PMC9295606

[48] Sudeshika T Naunton M Deeks LS , et al. General practice pharmacists in Australia: a systematic review. PLoS One 2021; 16: e0258674. 34648595 10.1371/journal.pone.0258674 PMC8516208

[49] Harding A What is the difference between an impact and an outcome? Impact is the longer term effect of an outcome. London, England: London School of Economics and Political Science, 2014, https://blogs.lse.ac.uk/impactofsocialsciences/2014/10/27/impact-vs-outcome-harding/ (accessed 26 May 2020).

[50] Hariton E Locascio JJ . Randomised controlled trials - the gold standard for effectiveness research. Bjog Bjog-Int J Obstet Gy 2018; 125: 1716–1716. 10.1111/1471-0528.15199 PMC6235704 29916205

[51] Perez-Gomez A Mejia-Trujillo J Mejia A . How useful are randomized controlled trials in a rapidly changing world? Glob Ment Health 2016; 3: e6. 10.1017/gmh.2015.29 PMC5314745 28596875

[52] Monti S Grosso V Todoerti M , et al. Randomized controlled trials and real-world data: differences and similarities to untangle literature data. Rheumatology 2018; 57: vii54–vii58. 30289534 10.1093/rheumatology/key109

[53] Rigby S . How to ensure effective employee appraisals. First Practice Management: Leeds. 2021, https://www.firstpracticemanagement.co.uk/blog/2021-blog-posts/how-to-ensure-effective-employee-appraisals/ (accessed 20 April 2023).

[54] Campbell R . Practice staff appraisals. GP practice manager (UK), 2018, http://gpsurgerymanager.co.uk/practice-staff-appraisals/ (accessed 20 April 2023).

