# Supplementary material for: Integration and impact of pharmacists in general practice internationally: A rapid review
Source: J Health Serv Res Policy. 2023 Jun 17;29(1):56–67. doi: 10.1177/13558196231179831 (PMC10729538; doi:10.1177/13558196231179831)
Supplement: Supplemental Material - Integration and impact of pharmacists in general practice internationally: A rapid review [file sj-pdf-1-hsr-10.1177_13558196231179831.pdf]

Journal of Health Services Research & Policy
Integration and impact of pharmacists in general practice internationally: a rapid review
Karampatakis GD, Patel N, Stretch G, and Ryan K

S1: Search strategies employed for the databases searched for this rapid review

S1 presents the precise search strategy for each database that was searched to identify relevant literature for this rapid review.

	**Search strategy**
**PubMed** (last search on 11/06/2021)	(pharmacists[Title/Abstract] OR pharmacist[Title/Abstract]) AND (("general practice"[MeSH Terms] OR ("general"[All Fields] AND "practice"[All Fields]) OR "general practice"[All Fields]) OR ("family practice"[MeSH Terms] OR ("family"[All Fields] AND "practice"[All Fields]) OR "family practice"[All Fields]))
**Web of Science** (last search on 11/06/2021)	#2 AND #1, where #1: TS=(pharmacist OR pharmacists) and #2: TS=(general practice OR family practice)

S2: Data extraction table, summarising the studies included in this rapid review

S2 presents the data extraction table that summarises all studies included in this rapid review. It also lists the enumeration of the references. The full list of references is given at the end of S2.

Author (year), country (reference number)	Study type and aim	Study population	Programme of integrating pharmacists into general practice	Activities of general practice-based pharmacists (GPBPs)	Details on GPBP impact identification methods used	Key findings of the study (relevant to the scope of this review)
Alshehri et al. (2021), UK (1)	Cross-sectional survey assessing GPBP role and integration.	GPBPs in England, regardless of integration scheme.	Nationwide governmental scheme and various other efforts (see also 'key findings' column).	See 'key findings' column.	There was no information on this matter.	Majority of GPBPs in general practice since 2015; 81% of GPBPs were independent prescribers (IPs); most had previous experience in community; 52% part-time employment, the rest full-time; 54% one practice, the rest multiple practices; main activities: medicines reconciliations, telephone consultations, face-to-face medication reviews.
Baker et al. (2019), Australia (2)	Mixed-methods study investigating roles, facilitators and barriers, remuneration of GPBPs.	Pharmacists accredited for home medication reviews and GPBPs.	There was no information on this matter.	See 'key findings' column.	There was no information on this matter.	No common funding model; 1 to 5 days/week in practices; concomitant affiliations; roles: medication reviews (in practices or homes), adherence ascertainment, patient counselling, de-prescribing, smoking cessation, medicines reconciliations, care liaison, staff education, audits.
Banh et al. (2021), Canada (3)	Descriptive study evaluating collaboration between GPBPs and general practitioners (GPs) and GPBP workload.	GPBPs, patients with at least one interaction with the GPBP.	Local effort in Edmonton, Alberta: 1 practice, part of the local primary care network (PCN); 1 GPBP, staff member of the local University; part-time employment; GPBP salary paid by local University; patient identification by GPBP and GPs; only adult patients seen by GPBP.	Medication reviews; long-term care; GP-approved care plans; monitoring; managing repeat prescription service; no clinical exams.	Quantifying number of patient encounters and GPBP activities.	Not applicable, as the quantity of GPBP activities and patient encounters were not viewed as outcomes to report in this review.
Benson et al. (2018), Australia (4)	Observational study describing-analysing recommendations of GPBP to GPs.	Patients at risk of medication misadventure and/or with medication assistance needs.	Went West General Practice Pharmacist Project, in Sydney (since 2016): funded by local Primary Health Network (PHN[a]); 13 practices; 4 GPBPs (recruited by PHN and practices); part-time employment mainly; coverage of multiple practices.	Face-to-face medication reviews; patient counselling; lifestyle advice.	Measurement of volume of GPBP recommendations and percentage of those actioned.	88% of GPBP recommendations actioned by GPs.

Author (year), country (reference number)	Study type and aim	Study population	Programme of integrating pharmacists into general practice	Activities of general practice-based pharmacists (GPBPs)	Details on GPBP impact identification methods used	Key findings of the study (relevant to the scope of this review)
Benson et al. (2018), Australia (5)	Observational study exploring detection and resolution of medication-related problems (MRPs) by GPBPs.	Patients with complex medication regimes and/or multiple co-morbidities.	Went West General Practice Pharmacist Project: see ref 4, plus absence of common funding; patient identification: referrals, including patient self-referrals.	See ref 4 (same activities reported).	MRPs spotted and volume of GPBP recommendations; acceptance rates of recommendations.	1,124 MRPs detected; 70% of GPBP recommendations actioned by GPs.
Benson et al. (2018), Australia (6)	Process evaluation (mixed methods design) examining the implementation of GPBP services.	GPBPs, GPs, patients with polypharmacy and other MRPs.	Went West General Practice Pharmacist Project.	See ref 4 (same activities reported).	Quantification of GPBP activities and qualitative exploration of GP views.	Not applicable, as stakeholder views and quantity of GPBP activities were not viewed as outcomes of interest for this review.
Bishop et al. (2015), Canada (7)	Survey study exploring patient and GP satisfaction with GPBP services.	GPs, patients receiving GPBP-led anti-coagulation services.	Local scheme in Newfoundland and Labrador (2006): 1 practice, 1 GPBP.	Telephone consultations with anticoagulation patients, including counselling.	Survey exploring satisfaction of patients and GPs.	Not applicable, stakeholder views were not perceived as an outcome of interest for this review.
Bradley et al. (2018), UK (8)	Longitudinal survey study exploring role evolution and integration of GPBPs in England.	GPBPs integrated via phase 1 of the governmental scheme.	Nationwide, governmental scheme (this was a nationwide study).	See 'key findings' column.	There was no information on this matter.	Telephone consultations; triage; medicines reconciliation; face-to-face medication reviews; ordering tests; physical assessments; long-term/acute care; incentive programmes; prescribing including managing repeat prescriptions; producing policies; supporting patient groups; staff education; supervising students; liaison with community pharmacies.
Bruhn et al. (2013), UK (9)	Randomised controlled trial comparing GPBP-led medication	Patients on regular pain medications.	Pharmacist-led management of chronic pain in primary care (PIPPC) in 2010: 3 practices in East Anglia and 3 practices in Grampian.	Chronic pain, face-to-face medication reviews;	Collection of self-reported pain levels; life quality; general health and	Not applicable, as self-reported outcomes were not of interest for this review.

Author (year), country (reference number)	Study type and aim	Study population	Programme of integrating pharmacists into general practice	Activities of general practice-based pharmacists (GPBPs)	Details on GPBP impact identification methods used	Key findings of the study (relevant to the scope of this review)
	reviews with usual care.			prescribing duties; developing care plans.	anxiety; costs for hospital/practice visits, medications.	
Bungay et al. (2004), USA (10)	Observational study describing interactions between GPBPs and patients.	Patients with depression and/or dysthymia.	Local effort in Boston targeting depression and dysthymia patients (for 18 months): 9 practices; 5 GPBPs; part-time employment; pre-specified frequency for patient contact.	Depression medication reviews, over the telephone; monitoring; patient counselling.	Measuring volume of activities and patients; GPBP time spent with patients and GPs.	Not applicable, as the quantity of GPBP activities and duration of encounters were not amongst the outcomes of interest for this review.
Bush et al. (2018), UK (11)	Descriptive observational study to characterise range and volume of GPBP activity.	GPBPs practising in Dudley.	Prescribing and Medicines Management Function in Dudley (since 2002): funded by the local Clinical Commissioning Group (CCG[b]); 49 practices; 23 GPBPs; part-time employment.	See 'key findings' column.	Quantification of activities; assumptions about savings on GP time and costs.	Most common GPBP activities: medication reviews; long-term care; minor ailments; monitoring; medicine reconciliations; managing high-risk drugs and waste; prescribing duties; audits; facilitating campaigns; multidisciplinary discussions; incentive programmes.
Campbell et al. (2017), New Zealand (12)	Survey study exploring employment and roles of pharmacists in primary care.	Pharmacists working in primary care settings.	See 'key findings' column.	See 'key findings' column.	There was no information on this matter.	Pharmacy Action Plan, since 2017: 31 GPBPs; funding from various sources, not solely by practices; full-time and part-time employment; activities: staff education, dealing with practice staff queries, writing bulletins, audits, care coordination, medicine reconciliations, and medication reviews.
Cardwell et al. (2020), Ireland (13)	Non-randomised pilot study to explore costs and effectiveness of GPBP services.	Patients ≥ 65 years with polypharmacy.	Small, 6-month, nationwide pilot: 4 practices, 3 GPBPs, part-time employment, covering multiple practices, GPBPs recruited by research team, costs for room rental-GP time covered by study's budget, GPBPs at own room/reception office.	Note-based and face-to-face medication reviews; checking repeat prescriptions; staff education; dealing with staff queries;	Patient-reported data (e.g. healthcare use) before and after intervention; practice-level (e.g. medication changes), and cost	1,521 MRPs identified, 59.8% of de-prescribing problems actioned by GPs versus 5.8% of cost-related matters.

Author (year), country (reference number)	Study type and aim	Study population	Programme of integrating pharmacists into general practice	Activities of general practice-based pharmacists (GPBPs)	Details on GPBP impact identification methods used	Key findings of the study (relevant to the scope of this review)
				clinical audits; participation in staff meetings; no prescribing rights (GPs approved and implemented prescription changes).	data (cost-savings from medication changes, assuming pilot for 12 months); MRPs identified and proportion actioned by GPs.	
Cariveau et al. (2019), USA (14)	Single-cohort, before-after study evaluating a GPBP-led naloxone prescribing programme.	Patients on chronic opioid therapy for pain, for whom naloxone was indicated.	Local effort in Western North Carolina, aiming to optimise naloxone prescribing (mid-2016): 1 practice involved.	Facilitating naloxone prescribing; patient counselling; staff education.	Calculating prescribing rates of naloxone before and after GPBP-led intervention.	Higher rates post the intervention, statistically significant difference.
Carter et al. (2015), USA (15)	Prospective, cluster-randomized trial, with a control, comparing effectiveness of GPBP-led interventions with usual care.	Adult patients with uncontrolled blood pressure (BP).	Collaboration Among Pharmacists and physicians to Improve Outcomes Now (CAPTION), across 15 States (for 5 years): 32 practices involved, part of the local Patient Centred Medical Homes (PCMHs[c]).	Note-based and face-to-face hypertension medication reviews; patient counselling; lifestyle advice; following-up.	Calculating the proportion of patients achieving controlled BP; differences in BP reduction between study's arms.	Similar proportions achieving controlled BP; significantly higher BP reduction in intervention arm.
Carter et al. (2018), USA (16)	Prospective, cluster-randomized, controlled trial evaluating GPBP impact on guideline	Patients ≥ 50 years with cardiac risk factors (e.g. diabetes, hypertension, hypercholester	The Improved Cardiovascular Risk Reduction to Enhance Rural Primary Care (ICARE), in Iowa: 6 practices; 3 GPBPs; services provided virtually; patient identification by project facilitators.	Note-based medication reviews; medicines reconciliations.	Adherence to cardiac-related guidelines; certain clinical values. GPBP recommendations (in intervention	Significantly higher adherence in intervention arm; similar clinical values; 88.8% of GPBP recommendations accepted in full by GPs.

Author (year), country (reference number)	Study type and aim	Study population	Programme of integrating pharmacists into general practice	Activities of general practice-based pharmacists (GPBPs)	Details on GPBP impact identification methods used	Key findings of the study (relevant to the scope of this review)
	adherence and cardiovascular risk.	olemia).			arm) accepted.	
Castelli et al. (2018), USA (17)	Retrospective, descriptive analysis of patient electronic records to describe GPBP work.	Patients who have not achieved clinical goals of therapy; and GPBPs.	Successful Collaborative Relationships to Improve PatienT care (SCRIPT), in Pittsburgh (in 2009): 4 practices (part of PCMHs); 2 GPBPs; coverage of multiple practices.	Face-to-face medication reviews; patient counselling; staff education; care plans; telephone consultations; population health tasks (e.g. writing policies and protocols).	Calculating number of activities, patients encountered, MRPs identified.	9,375 MRPs were identified by GPBPs.
Chen et al. (2000), UK (18)	Qualitative study to understand content and feasibility of GPBP consultations.	GPBPs.	Local effort in South London (late 1990s): 3 pharmacists; patient identification: pharmacists or referrals including self-referrals.	Medication reviews (in practices or homes).	Audio-recoding consultations, transcripts were then thematically analysed.	Not applicable, as there were no quantifiable outcomes that would have been of interest for this review.
Cowart et al. (2019), USA (19)	Retrospective matched cohort study comparing the impact of a GPBP-led diabetes service with usual care.	Patients with type 2 diabetes mellitus.	Local, 12 month effort in Florida (since early 2014): 2 practices; 2 pharmacists; coverage of 1 practice; part-time employment.	Face-to-face medication reviews.	Time to treatment intensification; number of patients with reductions in glycated haemoglobin (HbA1c), those achieving HbA1c goals and time for this achievement; changes in HbA1c.	Positive differences, non-statistically significant though, favouring intervention arm.

Author (year), country (reference number)	Study type and aim	Study population	Programme of integrating pharmacists into general practice	Activities of general practice-based pharmacists (GPBPs)	Details on GPBP impact identification methods used	Key findings of the study (relevant to the scope of this review)
Croke et al. (2021), Ireland (20)	Protocol for a pilot cluster randomised controlled trial examining feasibility and impact of GPBP role as compared to usual care.	Patients ≥ 65 years on ≥ 10 regular medications, GPBPs, GPs, practice nurses and managers.	Small scale effort across Ireland (expected to last 4 months): 8 practices (both single-handed and group); 2 GPBPs, each covering 2 practices; part-time employment; GPBPs to be recruited by the research team through competitive interviews.	Medication reviews; audits; medicines reconciliations; staff queries and education sessions; long-term care; drug monitoring; telephone consultations; managing repeat prescription service; no prescribing rights.	Before-after design, comparators: inappropriate prescribing incidences/patient, medication-related (e.g. adverse effects) and patient-reported outcomes (e.g. life quality); quantity of activities and time invested; volume of liaison with practice staff-community pharmacists; economic: GPBP costs vs savings (inflamed to 12 months) and mixed-methods process evaluation: stakeholder views.	Not applicable, this was a protocol.
Deeks et al. (2018), Australia (21)	Pilot study evaluating GPBP role in asthma management.	GPBPs, GPs, and asthmatic patients.	Local, 12 month pilot in Canberra (between 2016 and 2017): funded by local PHN; 3 practices; 5 GPBPs (recruited by practices); part-time employment; flexible roles; patient identification: GPBPs and/or referrals.	See 'key findings' column.	Analysis of activity diaries: quantify activities-monitor changes in Asthma Control Test (ACT[d]); stakeholder interviews (views on GPBP services).	Most common GPBP activities: asthma management, including asthma medication reviews; patient counselling; developing care plans.

Author (year), country (reference number)	Study type and aim	Study population	Programme of integrating pharmacists into general practice	Activities of general practice-based pharmacists (GPBPs)	Details on GPBP impact identification methods used	Key findings of the study (relevant to the scope of this review)
Deeks et al. (2018), Australia (22)	Mixed-methods study exploring perceptions about the role and integration of GPBPs.	Patients, practice staff, community pharmacists interacting with GPBPs.	Local, 12 month pilot in Canberra (see ref 21).	Medication reviews; patient counselling; lifestyle advice; audits; staff education; dealing with practice staff queries; administrative and research duties.	Exploration of stakeholder views via questionnaires and semi-structured interviews.	Not applicable, as stakeholder views were not amongst the outcomes to report in this review.
Deeks et al. (2018), Australia (23)	Pilot trial to describe GPBP activities.	GPBPs.	Local, 12 month pilot in Canberra (see ref 21).	See ref 21 and ref 22.	Analysis of activity diaries, using evolving coding systems, to quantify GPBP activities.	This study revealed the same activities as in ref 21 and ref 22 (see above).
Farrell et al. (2010), Canada (24)	Survey evaluating GP perceptions about GPBPs (during GPBP integration).	GPs at practices with GPBPs integrated.	Integrating Family Medicine and Pharmacy to Advance Primary Care Therapeutics (IMPACT), in Ontario (between 2003 and 2006): 7 practices; 7 GPBPs; governmental funds; practices and GPBPs recruited by research team.	Face-to-face medication reviews; ongoing patient following-up; monitoring; staff education; quality assurance projects.	GPs comparing own and GPBP contributions to practices (questionnaires at 3 different time points).	Not applicable, as there were no independently measured, quantifiable outcomes that would have been of interest for this review.
Farrell et al. (2013), Canada (25)	Qualitative, ethnographic study exploring evolving GPBP routines and GPBP/other provider views of GPBP roles.	GPBPs, practice staff and patients.	IMPACT and Family Health Team Initiative.	See ‘key findings’ column.	There was no information on this matter.	Most common GPBP activities: dealing with practice staff queries; direct patient care including care plans; medication use projects.

Author (year), country (reference number)	Study type and aim	Study population	Programme of integrating pharmacists into general practice	Activities of general practice-based pharmacists (GPBPs)	Details on GPBP impact identification methods used	Key findings of the study (relevant to the scope of this review)
Freeman et al. (2012), Australia (26)	Retrospective analysis determining GPBP impact on medication review timeliness and completion.	The GPBP and patients having consulted the GPBP.	Local scheme in Brisbane (early 2009): 1 practice; 1 GPBP; patient identification: via referrals.	Face-to-face medication reviews (in practices or homes); staff education; patient counselling.	Before-after design: number of patients referred for medication review; time to review completion (from referral); financial impact on practices.	Less time to complete reviews and financial savings due to more reviews completed hence billed, unclear statistical significance.
Gillespie et al. (2017), Canada (27)	Survey exploring the focus of GPBP activities.	GPBPs practising in Ontario.	IMPACT and Family Health Team Initiative, the latter since 2006: 111 practices (out of the 190 in Ontario); 155 GPBPs, more than 1/2 covered multiple practices; full-time in practices for 1/3 of cases.	See 'key findings' column.	Impact on patients, as perceived by GPBPs in the survey.	Most common activities: patient counselling; clinical medication reviews; medicine reconciliations; staff education; dealing with practice staff queries; student supervision.
Guénette et al. (2020), Canada (28)	Cross-sectional survey describing GPBP characteristics and employment.	GPBPs practising in Quebec.	Governmental scheme in Quebec (see 'key findings' column).	See 'key findings' column.	There was no information on this matter.	Direct, part-time, employment by practices; 1 GPBP/practice; most working in community too; GPBPs organised in networks for training and research purposes; main activities: medication reviews; de-prescribing; telephone consultations with patients; dealing with practice staff queries.
Gums et al. (2014), USA (29)	Prospective before-after study examining if GPBP-led services improve asthma control.	Patients ≥ 12 years with persistent asthma.	CAPTION (see ref 15).	Face-to-face, asthma medication reviews; telephone support; patient counselling.	Before-after design: hospitalisations and Accident & Emergency (A&E) visits; ACT scores; self-reported life quality.	Significantly fewer hospitalisations and A&E visits, deterioration when scheme discontinued.
Gums et al. (2015),	Prospective, cluster-	Adults on anti-hypertensive	CAPTION (see ref 15).	See ref 15, plus adherence	Comparing: self-reported medication	Intervention arm: significantly higher treatment intensification (i.e. significantly more

Author (year), country (reference number)	Study type and aim	Study population	Programme of integrating pharmacists into general practice	Activities of general practice-based pharmacists (GPBPs)	Details on GPBP impact identification methods used	Key findings of the study (relevant to the scope of this review)
USA (30)	randomized trial (see ref 15).	treatment.		ascertainment, care plans for approval by GPs.	adherence; medication changes and use.	medication changes, significantly increased use of diuretics and aldosterone antagonists).
Haag et al. (2021), USA (31)	Cross-sectional survey study exploring perceived GPBP impact on practice staff.	GPs, practice nurses and physician assistants.	Efforts in 4 regions across 2 states: 16 GPBPs; part-time employment.	Medication reviews; prescribing duties; drug monitoring, including ordering laboratory tests.	Practice staff views: impact on workload and capacity; meaning in work; overall satisfaction with GPBPs.	Not applicable, as self-reported outcomes were not of interest for this review.
Hall et al. (2009), USA (32)	Retrospective chart review evaluating GPBP impact on osteoporosis treatment.	Adult patients with osteoporosis.	Local effort in South Carolina targeting osteoporosis patients (late 2008): funds from local university; 2 practices; 4 GPBPs.	Osteoporosis, face-to-face medication reviews; patient counselling; lifestyle advice; monitoring; test ordering.	Before-after design: adherence to osteoporosis guidelines.	Significantly higher adherence to guidelines after GPBP interventions.
Hampson et al. (2019), UK (33)	Qualitative interview study exploring the value GPs place on GPBP role.	GPs employing GPBPs, without using governmental funds.	GPBPs integrated into practices outside of the governmental schemes (in Midlands and East region of England).	There was no information on this matter.	See ‘key findings’ column.	Absence of a common impact identification process for GPBPs, different approaches by different practices.
Harris et al. (2009), USA (34)	Prospective, observational, cohort study evaluating impact of GPBP-led services on care quality.	Patients at risk of MRPs.	Local effort in Minnesota (in 2000): 1 practice (university-affiliated); patient identification: by GPBPs.	Face-to-face medication reviews; following-up.	MRPs resolved; changes in certain conditions’ status, use of certain drugs, number of drugs/patient, achievement of clinical goals.	250 MRPs spotted and resolved by GPBPs; statistically significant changes in all measures after GPBP intervention, except for number of patients achieving clinical goals.

Author (year), country (reference number)	Study type and aim	Study population	Programme of integrating pharmacists into general practice	Activities of general practice-based pharmacists (GPBPs)	Details on GPBP impact identification methods used	Key findings of the study (relevant to the scope of this review)
Haua et al. (2019), New Zealand (35)	Survey study to characterise landscape with GPBPs in New Zealand.	GPBPs.	See 'key findings' column.	See 'key findings' column.	There was no information on this matter.	36 GPBPs, 104 practices; 14 GPBPs in 1 practice, the rest multiple practices; GPBPs concentrated in certain regions; 66% full-time employment; funding mostly from Primary Health Organisations (PHOs[e]) and District Health Boards (DHBs[f]); main activities: medication reviews, long-term condition management and medicines reconciliations.
Hazen et al. (2019), Netherlands (36)	Theory evaluation exploring what it is entailed in integrating pharmacists into general practices.	GPBPs.	Pharmacotherapy Optimisation through Integration of a Non-dispensing pharmacist in a primary care Team (POINT), in Utrecht and Amsterdam (from early 2014 and for 15 months): 10 practices; 10 GPBPs; full-time employment; a fixed income for GPBPs; patient identification: by GPBPs, and via referrals or self-referrals.	Patient care tasks (e.g. medication reviews); quality management tasks (e.g. medication use projects); medicines reconciliation; practice staff education.	Ethnographic methods to identify pharmacist impact on medication safety as mirrored by their interactions with professionals.	Not applicable, as there were no independently measured, quantifiable outcomes that would have been of interest for this review.
Hazen et al. (2019), Netherlands (37)	Observational cross-sectional study exploring GPBP impact on MRPs.	Elderly patients with polypharmacy.	POINT (see ref 36).	See ref 36, plus care plans.	MRPs spotted; pharmacists' recommendations and proportion actioned; proportion of MRPs resolved.	1292 MRPs; 83% of recommendations actioned; 78% of MRPs resolved.
Hill et al. (2019), UK (38)	Descriptive study looking into the implementation of GPBP services as a means to tackle opioid analgesic	Patients at pain-related conditions receiving opioid analgesic	Local scheme in Lanarkshire, aiming to reduce opioid prescribing (late 2015): 2 practices; 2 pharmacists (from local addiction services); part-time employment.	Opioid medication reviews; prescribing duties.	Volume of records reviewed and patient contact; volume of opioid analgesics prescribed before	Volume of opioid analgesics reduced after GPBP interventions, unclear statistical significance.

Author (year), country (reference number)	Study type and aim	Study population	Programme of integrating pharmacists into general practice	Activities of general practice-based pharmacists (GPBPs)	Details on GPBP impact identification methods used	Key findings of the study (relevant to the scope of this review)
	dependence.	medication.			and after integration of pharmacists.	
Isetts et al. (2016), USA (39)	Descriptive analysis to understand the components of GPBP work in the management of hypertension.	GPBPs.	CAPTION (see ref 15), plus part-time employment; funding in some practices from billing patients.	See ref 15 and ref 30, plus education to practice staff.	Calculating number of encounters with patients; pharmacists' time on certain activities.	Not applicable, as the quantity and duration of GPBP activities were not amongst outcomes of interest for this review.
James et al. (2020), Ireland (40)	Qualitative process evaluation exploring the implementation of GPBP services.	GPBPs, patients, GPs and other practice staff.	See ref 13, plus 2 GPBPs exclusively in one practice, 1 covering 2 practices.	See ref 13 and 'key findings' column.	There was no information on this matter. .	One additional GPBP activity: care liaison with pharmacists in hospital and community.
Jun (2018), USA (41)	Descriptive study to understand the development and implementation of GPBP services.	GPBPs, patients having contacted a GPBP.	Local effort in Southern California (mid-2014): funded by local university and a grant; 1 practice (university-affiliated); 2 GPBPs; part-time employment; patient identification by referrals.	Face-to-face medications reviews; patient counselling; managing repeat prescription service; practice staff education.	Number of patients interacted with pharmacists; hours spent with patients; changes in HbA1c.	Reduction in HbA1c, unclear statistical significance.
Karampatakis et al. (2019), UK (42)	e-Delphi study aiming to reach consensus on GPBP activities should be recorded to identify impact.	GPBPs and practice pharmacy technicians.	Nationwide governmental efforts (West London area).	See 'key findings' column.	Key performance indicators, at a national level, requiring GPBPs to record work using electronic codes.	Main activities reported: medication reviews at practices, homes, nursing homes; diagnostic elements; prescribing duties, and overseeing repeat prescription service; monitoring high risk drugs; medicines reconciliations; staff education-queries; leading team meetings; audits; telephone consultations and triage;

Author (year), country (reference number)	Study type and aim	Study population	Programme of integrating pharmacists into general practice	Activities of general practice-based pharmacists (GPBPs)	Details on GPBP impact identification methods used	Key findings of the study (relevant to the scope of this review)
						supporting patient participation groups.
Karampatakis et al. (2019), UK (43)	Qualitative study exploring impact measurement problems of GPBPs.	GPBPs, GPs, practice managers.	Nationwide governmental efforts (West London area).	See ref 42.	See ref 42 and 'key findings' column.	National measures not fit for purpose in demonstrating GPBP impact (not targeting GPBP work, not accounting for quality, and not capturing the depth and breadth of GPBP tasks).
Karampatakis et al. (2020), UK (44)	Qualitative study exploring community pharmacy team experiences of GPBPs.	Community pharmacists and pharmacy technicians.	Nationwide governmental efforts (West London area).	There was no information on this matter.	Community pharmacy team views on GPBP impact on them (via semi-structured interviews).	Not applicable, as self-reported outcomes were not of interest for this review.
Karampatakis et al. (2021), UK (45)	Qualitative study exploring patient experiences of GPBPs.	Adult patients having consulted a GPBP.	Nationwide governmental efforts (West London, Surrey and Berkshire areas).	There was no information on this matter.	Impact on patients, reported by patients (via semi-structured interviews).	Not applicable, as self-reported outcomes were not of interest for this review.
Kosari et al. (2021), Australia (46)	Prospective observational study assessing feasibility of determining cost-benefits of GPBPs.	GPBPs.	Study related to a local effort in Canberra (see ref 21); Workforce Incentive Programme: new governmental effort, since early 2020, contributing to expenses for employing clinicians; less than 100 GPBPs in Australia (mainly part-time employment).	No prescribing duties, see also 'key findings' column.	Analysing GPBP diaries: time spent on activities (to extrapolate time-savings for GPs), volume of income-generating tasks; value-cost models estimating income brought by GPBPs (based on several assumptions).	Main activities: medication reviews, medicines reconciliations, patient counselling, drug monitoring, care liaison (community pharmacies, care homes), smoking cessation, and following-up patients.
Langran et al. (2017), UK (47)	Uncontrolled prospective cohort study	Patients with type 2 diabetes.	Local, 12 month scheme in Slough (between 2013 and 2014): Commissioned by local CCG; 13	Face-to-face medication reviews for type 2	Before-after design: patients receiving care following	Positive changes, deteriorating after scheme's discontinuation, unclear statistical significance.

Author (year), country (reference number)	Study type and aim	Study population	Programme of integrating pharmacists into general practice	Activities of general practice-based pharmacists (GPBPs)	Details on GPBP impact identification methods used	Key findings of the study (relevant to the scope of this review)
	assessing GPBP impact on type 2 diabetes management and control.		practices; 4 GPBPs (employed by CCG but fully integrated); coverage of multiple practices; patients identified by GPBPs.	diabetes; lifestyle advice.	guidelines; those having certain clinical values outside range.	
MacRae et al. (2003), UK (48)	Survey study ascertaining GP views of GPBP services.	GPs from practices with GPBPs integrated.	Local, 2-year scheme in Glasgow (from late 1999): funded by the local Primary Care Trust[g]; 82 practices; 27 GPBPs; part-time employment.	Face-to-face medication reviews.	Questionnaire exploring GPBP impact on GPs, as perceived by GPs.	Not applicable, as self-reported outcomes were not of interest for this review.
Marques et al. (2018), UK (49)	Mixed-methods study exploring matters with integration of pharmacists into general practice and perceived impact of GPBPs on care delivery and community pharmacy practice.	GPBPs, community pharmacists, scheme commissioners and patients with GPBP contact.	Primary Care Pharmacy Programme (PCPP), in Sheffield (between 2015 and 2017): see also 'key findings' column.	Medicines reconciliations; medication reviews (note-based or face-to-face in practices or care homes); prescription queries from patients and pharmacies.	Quantifying activities; perceived estimations on time-savings for GPs; exploring patient and community pharmacist views (via surveys and interviews, respectively).	86 practices participated in PCPP (of which 9 continued to employ a pharmacist post the scheme); part-time employment for GPBPs.
Maskrey et al. (2018), UK (50)	Prospective observational cohort study assessing GPBP impact on GPs.	GPs having a GPBP in their practice, other practice staff, and GPBPs.	Nationwide, governmental scheme (Glasgow area): 16 practices, part of the local Health and Social Care Partnership (HSCP[h]); roles developed collectively by HSCP lead and practices.	Prescribing tasks; medicines reconciliation; queries from practice staff and pharmacies.	Before-after design: GP time on certain prescribing tasks; exploring GP experiences (via a survey).	GP time reduced, unclear statistical significance.
Moczygemba et al. (2019), USA (51)	Retrospective, cross-sectional, controlled study exploring GPBP	Patients with chronic conditions.	Local effort in Stuart, Florida: 4 practices; part-time employment; patients identification by referrals.	Face-to-face medication reviews and medicines	Before-after design, comparators: unplanned hospital admissions, A&E	Hospital admissions decreased in intervention and increased in control (not significantly); A&E visits stable in intervention but increased significantly in control.

Author (year), country (reference number)	Study type and aim	Study population	Programme of integrating pharmacists into general practice	Activities of general practice-based pharmacists (GPBPs)	Details on GPBP impact identification methods used	Key findings of the study (relevant to the scope of this review)
	impact on healthcare use and costs.			reconciliations; monitoring; patient counselling; care plans.	visits, benefit-cost ratio (GPBP direct costs vs literature-based savings); quantifying activities.	
Nabhani-Gebara et al. (2020), UK (52)	Exploratory multiple case study exploring dynamics in practices following GPBP integration.	GPBPs, practice nurses, GPs, and patients who consulted a GPBP.	Efforts in South-East of England, outside nationwide governmental attempts.	There was no information on this matter.	Identification of changes in the dynamics amongst professionals (via in-depth qualitative interviews); eliciting patient satisfaction (via a survey).	Not applicable, as self-reported outcomes were not of interest for this review.
Neilson et al. (2015), UK (53)	Regression analysis of costs and effects, as part of a randomised controlled trial (also see ref 9).	Patients on regular pain medications.	PIPPC (see ref 9).	See ref 9.	Differences, between control and intervention arm, in mean total costs (using intention-to-treat models) and effects (using self-reported life quality).	Not applicable, as self-reported outcomes and costs based on assumptions were not of interest for this review.
Nelson et al. (2019), UK (54)	Qualitative study comparing the establishment of 3 non-medical roles in general practice.	Service and training leads, practice nurses, physician associates and managers, GPs, GPBPs.	Nationwide, governmental scheme (Manchester area).	No relevant information on this matter.	See ‘key findings’ column.	Difficulties in measuring differences on workload of GPs.

Author (year), country (reference number)	Study type and aim	Study population	Programme of integrating pharmacists into general practice	Activities of general practice-based pharmacists (GPBPs)	Details on GPBP impact identification methods used	Key findings of the study (relevant to the scope of this review)
Odenthal et al. (2020), USA (55)	Retrospective chart review to evaluate GPBP impact on de-prescribing.	Adults on long-term proton pump inhibitors for gastroesophageal reflux.	Local effort in St. Paul (Minnesota): 2 GPBPs; 1 practice; patients identified by GPBPs.	Care plans involving tapering instructions; patient counselling and following-up.	Calculating de-prescribing rates in relation to proton pump inhibitors.	Successful de-prescribing: vast majority of patients having consulted a GPBP completely discontinued or decreased dose/frequency of proton pump inhibitors.
Petty et al. (2003), UK (56)	Qualitative study ascertaining patient views of GPBP-led medication reviews.	Patients ≥ 65 years who consulted a GPBP.	Local scheme in Leeds (late 1990s).	Face-to-face medication reviews.	Focus group exploring patient views.	Not applicable, as self-reported outcomes were not of interest for this review.
Pottie et al. (2008), Canada (57)	Qualitative study exploring GP views on collaboration with GPBPs.	GPs having a GPBP in their practice.	IMPACT (see ref 24).	See ref 24 and ref 25.	Focus groups and interviews exploring GP views of GPBP impact.	Not applicable, as self-reported outcomes were not of interest for this review.
Pottie et al. (2009), Canada (58)	Qualitative analysis of GPBP narrative accounts to understand identity development.	GPBPs in IMPACT.	IMPACT (see ref 24), plus part-time employment; concomitant affiliations.	See ref 24 and ref 25.	There was no information on this matter.	Findings on identity development were outside the scope of this review.
Prudencio et al. (2020), USA (59)	Retrospective chart review determining GPBP impact on patient outcomes.	Patients with type 1 or type 2 diabetes who had consulted a GPBP.	Local effort (since 2016) in Hilo, Hawaii: 1 practice affiliated with local University; 2 GPBPs (academic members of the University); part-time employment; funding from local University; patient identification via referrals and by GPBPs; varied GPBP	Face-to-face medication reviews; patient counselling; lifestyle advice; following-up, including ordering	Before-after design: composite of goal attainment rates, individual goal attainment rates (goals: HbA1c, BP, appropriate statin	Significant improvements both in the composite and individual goals attainment rates after intervention.

Author (year), country (reference number)	Study type and aim	Study population	Programme of integrating pharmacists into general practice	Activities of general practice-based pharmacists (GPBPs)	Details on GPBP impact identification methods used	Key findings of the study (relevant to the scope of this review)
			models in terms of integration, practice and funding across the USA, due to different state regulations.	laboratory tests; medicines reconciliations.	therapy).	
Rodgers et al. (1999), UK (60)	Controlled trial evaluating whether implantation of GPGP services results in savings for practices.	Patients with long-term conditions.	Local, 12 month scheme in Doncaster (in 1996): funded by local Health Authority[i]; 8 practices; 5 pharmacists.	Switches to generics; audits; reviewing repeat prescribing; formulary reviews; asthma/gastrointestinal medication reviews.	Calculating changes in prescribing costs; whether savings offset scheme's costs.	Significantly fewer prescribing costs in intervention arm, which offset scheme's costs.
Rothman et al. (2003), USA (61)	Evaluation of GPBP-led diabetes management programme to determine impact on HbA1c control.	Patients with type 2 diabetes and poor glycose control.	Local effort in North Carolina targeting diabetes patients (in 1999): 1 practice (university-affiliated); 3 GPBPs.	Diabetes medication review; patient counselling; patient following-up; elements of physical assessment; care plans.	Before-after design: HbA1c.	Statistically significant improvements.
Ryan et al. (2018), UK (62)	Exploratory qualitative study investigating practicalities of setting up and maintaining GPBP services.	GPBPs, patients who consulted a GPBP, GPs, clinical and administrative practice staff.	Nationwide, governmental scheme (West London area): local effort pre-dated nationwide scheme (GP Federation[j] hiring a private company to integrate pharmacists); 8 practices; patient identification: referrals including self-referrals.	See 'key findings' column.	Exploration of stakeholder views (via in-depth interviews).	Most common activities: face-to-face medication reviews (in practices or care homes); long-term/acute care; monitoring; spirometry; triage; medicines reconciliation; audits and incentive programmes; prescribing including authorising repeat prescriptions.
Savickas et al. (2021), UK (63)	Cross-sectional survey study exploring GPBP services and role	GPBPs and general practice-based pharmacy	Nationwide study, referring to governmental and non-governmental schemes.	See 'key findings' column.	Benefits for patients and practices, as perceived by participants.	76.5% of GPBPs IPs; most covering 'too many practices'; main activities: medication reviews, management of polypharmacy-long-term conditions, medicines reconciliations, audits;

Author (year), country (reference number)	Study type and aim	Study population	Programme of integrating pharmacists into general practice	Activities of general practice-based pharmacists (GPBPs)	Details on GPBP impact identification methods used	Key findings of the study (relevant to the scope of this review)
	development.	technicians.				GPBPs integrated through governmental schemes: significantly more likely to manage repeat prescription service.
Slazak et al. (2020), USA (64)	Pilot study describing implementation of GPBP services and evaluating impact.	Patients with hospital discharges.	Local effort in Western New York: 2 practices; 3 GPBPs; full-time employment; patient identification by practice nurses.	Face-to-face or telephone medicines reconciliations and medication reviews; GP-approved care plans; patient counselling.	Volume of GPBP recommendations and percentage actioned; comparisons between intervention-usual care: 30-day all-cause hospital readmissions.	Approximately 80% of GPBP recommendations were actioned by GPs; no significant difference, between study's arms, in hospital readmissions within 30 days from an earlier discharge.
Sloeserwij et al. (2019), Netherlands (65)	Non-randomised controlled intervention study exploring GPBP impact on pharmacotherapy safety.	Patients with multi-morbidity and/or polypharmacy.	POINT (see ref 36).	See ref 36 and ref 37.	Before-after design: medication-related hospital admissions; drug burden index[k]; costs for medication, hospital care, tests.	Fewer hospitalisations in intervention arm, unclear statistical significance; no difference on drug burden index and costs.
Sloeserwij et al. (2020), Netherlands (66)	Non-randomised, controlled intervention study (see ref 65).	Patients with multi-morbidity and/or polypharmacy.	POINT (see ref 36).	See ref 36 and ref 37.	Before-after design: GP prescribing quality (use of 10 indicators and patient records).	No consistent effect on GP prescribing quality (when compared to control arm, difference only on indicator around monitoring renal function in antihypertensive therapies).
Stewart et al. (2020), UK (67)	Cross-sectional survey to characterise pharmacy workforce in	Pharmacists and pharmacy technicians in Scottish general	Nationwide, governmental scheme (study was carried out across Scotland), see 'key findings' column.	See 'key findings' column.	There was no information on this matter.	Coverage of 2 practices/GPBP; common parallel affiliations; about 70% of GPBPs were IPs; main activities: clinical duties such as medication reviews; population level duties such as audits and care coordination.

Author (year), country (reference number)	Study type and aim	Study population	Programme of integrating pharmacists into general practice	Activities of general practice-based pharmacists (GPBPs)	Details on GPBP impact identification methods used	Key findings of the study (relevant to the scope of this review)
	Scotland.	practices.				
Sudeshika et al. (2021), Australia (68)	Protocol for a cross-national mixed-methods study exploring inter-professional collaboration after GPBP introduction.	GPBPs, GPs and other practice staff.	New pilot in Canberra for 18 months: 8 practices; part-time employment; GPBPs to be recruited by practices; funding by local PHN.	Medication reviews; clinical audits; patient counselling; practice staff education and queries.	Longitudinal surveys and semi-structured interviews to identify changes in inter-professional collaboration and team effectiveness.	Not applicable, this was a protocol.
Syafhan et al. (2021), UK (69)	Multi-centre, randomised controlled trial evaluating GPBP impact on patient outcomes and healthcare costs.	Adult patients at risk of MRPs.	Governmental schemes in England and Northern Ireland (study was carried out in four different geographical locations); patient identification via self-referrals (patients invited via flyers).	Face-to-face medication reviews, adherence ascertainment; patient counselling; lifestyle advice; referrals to other professionals; care plans; following up.	Before-after design, comparators: MRPs, medication appropriateness, unplanned hospital admissions, A&E visits, practice consultations, outpatient visits, costs savings due to healthcare utilisation, self-reported life quality-medication beliefs-adherence; patient satisfaction overall.	Significant decrease in median numbers of MRPs/patient and telephone consultations with practice staff in intervention arm; significant improvement in medication appropriateness in intervention arm; no significant differences, between arms, in unplanned hospital admissions, length of hospital stay, A&E attendances, outpatient visits; cost savings due to healthcare utilisation in intervention arm (not statistically significant reduction).
Tan et al. (2013), Australia (70)	Qualitative study eliciting views on GPBP services.	GPBPs, patients, GPs, practice nurses and managers.	The Pharmacists in Practice Study (PIPS), in Melbourne (between 2011 and 2013): funded by the research team; 2 practices; 2 GPBPs; part-time employment; patient identification: via referrals.	Face-to-face medication reviews; adherence ascertainment; patient	Exploring views of patients and practice staff.	Not applicable, as stakeholder views were not perceived as an outcome to report in this review.

Author (year), country (reference number)	Study type and aim	Study population	Programme of integrating pharmacists into general practice	Activities of general practice-based pharmacists (GPBPs)	Details on GPBP impact identification methods used	Key findings of the study (relevant to the scope of this review)
				counselling; lifestyle advice.		
Tan et al. (2014), Australia (71)	Evaluation examining GPBP impact on osteoporosis management.	Patients ≥ 50 years with established osteoporosis diagnosis.	PIPS (see ref 70).	Osteoporosis audit; staff education; patient counselling.	Before-after design: number of patients on osteoporosis medications, vitamin D and/or calcium.	Significantly more patients on all three aspects following GPBP interventions.
Tan et al. (2014), Australia (72)	Prospective intervention study evaluating GPBP consultation effectiveness.	Patients with risk factors for MRPs.	PIPS (see ref 70).	See ref 70.	Before-after design: number of MRPs; self-reported patient adherence, general health and satisfaction.	Fewer MRPs, statistical significance.
Vande Griend et al. (2014), USA (73)	Descriptive study exploring the clinical impact of a GPBP service.	Patients who would have benefited from GPBP contact, as determined by GPBP.	Local, 12 month effort in Colorado (in 2011): 1 GPBP; onsite and offsite employment.	Note-based medication reviews.	Number of records screened, MRPs, recommendations and acceptance rates, cost savings due to drug switches.	70% of GPBP recommendations actioned, 24 serious interactions resolved, savings of about $52,000.
Young et al. (2011), Canada (74)	Retrospective cohort study comparing GPBP-led anticoagulation management to that provided by GPs.	Patients on warfarin.	Local scheme in Newfoundland and Labrador (2006), see ref 7.	See ref 7.	Comparisons: times International Normalised Ratio (INR) within therapeutic and expanded therapeutic range (i.e. range ± 0.3); >5.0 or <1.5.	Statistically significant differences, favouring the intervention arm.
Zermansky	Randomised	Patients ≥ 65	Local scheme in Leeds (same as in ref	Face-to-face	Comparing:	More prescription changes, less medications

Author (year), country (reference number)	Study type and aim	Study population	Programme of integrating pharmacists into general practice	Activities of general practice-based pharmacists (GPBPs)	Details on GPBP impact identification methods used	Key findings of the study (relevant to the scope of this review)
et al. (2001), UK (75)	controlled trial comparing GPBP-led medication reviews with usual general practice reviews.	years receiving repeat prescriptions.	56).	medication reviews.	changes in repeat prescriptions; volume of medications prescribed and costs; hospital and practice use.	and costs in intervention arm (unclear statistical significance); similar use of hospital/practices.

[a]Australian Primary Health Networks have a similar function to the English Primary Care Networks.

[b]Clinical Commissioning Groups are legal bodies in the UK, part of the NHS, that design and commission healthcare services for their local area.

[c]Patient Centred Medical Homes are the US version of Primary Care Networks, promoting integrated care and enhanced cooperation between professionals and patients.

[d]Asthma Control Test is a patient questionnaire looking at asthma control over the last four weeks.

[e]Primary Health Organisations are structures in New Zealand primary care, funded by District Health Boards, that secure the provision of healthcare services via local general practices. They resemble to the UK PCNs.

[f]District Health Boards in New Zealand are responsible for the provision and funding of healthcare services in their region, they resemble to the UK CCGs.

[g]Primary Care Trusts were former structures in the Scottish primary care.

[h]Health and Social Care Partnerships are the Scottish version of the English Primary Care Networks.

[i]Health Authorities were administrative structures of NHS in the period between 1982 and 2000.

[j]A GP Federation was a former structure in the UK primary care setting which was composed of a number of general practices operating together as part of a collective union and within a certain geographical area.

[k]Drug burden index is an indicator mirroring the exposure of elderly to anticholinergic and sedative agents.

References

1. Alshehri AA, Cheema E, Yahyouche A, et al. Evaluating the role and integration of general practice pharmacists in England: a cross-sectional study. *Int J Clin Pharm*. Epub ahead of print 2 June 2021. DOI: 10.1007/s11096-021-01291-6.
2. Baker S, Lee YP, Hattingh HL. An evaluation of the role of practice pharmacists in Australia: a mixed methods study. *Int J Clin Pharm* 2019; 41: 504–515.
3. Banh HL, Cave AJ. A De Novo Pharmacist-Family Physician Collaboration Model in a Family Medicine Clinic in Alberta, Canada. *Pharmacy (Basel)* 2021; 9: 107.
4. Benson H, Lucas C, Benrimoj SI, et al. Pharmacists in general practice: recommendations resulting from team-based collaborative care. *Aust J Prim Health* 2018; 24: 448–454.
5. Benson H, Lucas C, Kmet W, et al. Pharmacists in general practice: a focus on drug-related problems. *Int J Clin Pharm* 2018; 40: 566–572.
6. Benson H, Sabater-Hernández D, Benrimoj SI, et al. Piloting the Integration of Non-Dispensing Pharmacists in the Australian General Practice Setting: A Process Evaluation. *Int J Integr Care* 2018; 18: 4.
7. Bishop L, Young S, Twells L, et al. Patients' and physicians' satisfaction with a pharmacist managed anticoagulation program in a family medicine clinic. *BMC Res Notes* 2015; 8: 233.
8. Bradley F, Seston E, Mannall C, et al. Evolution of the general practice pharmacist's role in England: a longitudinal study. *Br J Gen Pract* 2018; 68: e727–e734.

9. Bruhn H, Bond CM, Elliott AM, et al. Pharmacist-led management of chronic pain in primary care: results from a randomised controlled exploratory trial. *BMJ Open* 2013; 3: e002361.

10. Bungay KM, Adler DA, Rogers WH, et al. Description of a clinical pharmacist intervention administered to primary care patients with depression. *Gen Hosp Psychiatry* 2004; 26: 210–218.

11. Bush J, Langley CA, Jenkins D, et al. Clinical pharmacists in general practice: an initial evaluation of activity in one English primary care organisation. *Int J Pharm Pract* 2018; 26: 501–506.

12. Campbell C, Braund R, Morris C. Beyond the four walls: an exploratory survey of location, employment and roles of pharmacists in primary health care. *J Prim Health Care* 2017; 9: 297–310.

13. Cardwell K, Smith SM, Clyne B, et al. Evaluation of the General Practice Pharmacist (GPP) intervention to optimise prescribing in Irish primary care: a non-randomised pilot study. *BMJ Open* 2020; 10: e035087.

14. Cariveau D, Fay AE, Baker D, et al. Evaluation of a pharmacist-led naloxone coprescribing program in primary care. *J Am Pharm Assoc (2003)* 2019; 59: 867–871.

15. Carter BL, Coffey CS, Ardery G, et al. Cluster-randomized trial of a physician/pharmacist collaborative model to improve blood pressure control. *Circ Cardiovasc Qual Outcomes* 2015; 8: 235–243.

16. Carter BL, Levy B, Gryzlak B, et al. Cluster-Randomized Trial to Evaluate a Centralized Clinical Pharmacy Service in Private Family Medicine Offices. *Circ Cardiovasc Qual Outcomes* 2018; 11: e004188.

17. Castelli G, Bacci JL, Dombrowski SK, et al. Pharmacist-Delivered Comprehensive Medication Management Within Family Medicine Practices An Evaluation of the SCRIPT Project. *Fam Med* 2018; 50: 605–612.
18. Chen J, Britten N. “Strong medicine”: an analysis of pharmacist consultations in primary care. *Fam Pract* 2000; 17: 480–483.
19. Cowart K, Sando K. Pharmacist Impact on Treatment Intensification and Hemoglobin A1C in Patients With Type 2 Diabetes Mellitus at an Academic Health Center. *J Pharm Pract* 2019; 32: 648–654.
20. Croke A, Moriarty F, Boland F, et al. Integrating clinical pharmacists within general practice: protocol for a pilot cluster randomised controlled trial. *BMJ Open* 2021; 11: e041541.
21. Deeks LS, Kosari S, Boom K, et al. The Role of Pharmacists in General Practice in Asthma Management: A Pilot Study. *Pharmacy (Basel)* 2018; 6: 114.
22. Deeks LS, Kosari S, Naunton M, et al. Stakeholder perspectives about general practice pharmacists in the Australian Capital Territory: a qualitative pilot study. *Aust J Prim Health* 2018; 24: 263–272.
23. Deeks LS, Naunton M, Tay GH, et al. What can pharmacists do in general practice? A pilot trial. *Aust J Gen Pract* 2018; 47: 545–549.
24. Farrell B, Pottie K, Woodend K, et al. Shifts in expectations: evaluating physicians’ perceptions as pharmacists become integrated into family practice. *J Interprof Care* 2010; 24: 80–89.
25. Farrell B, Ward N, Dore N, et al. Working in interprofessional primary health care teams: what do pharmacists do? *Res Social Adm Pharm* 2013; 9: 288–301.

26. Freeman C, Cottrell WN, Kyle G, et al. Does a primary care practice pharmacist improve the timeliness and completion of medication management reviews? *Int J Pharm Pract* 2012; 20: 395–401.

27. Gillespie U, Dolovich L, Dahrouge S. Activities performed by pharmacists integrated in family health teams: Results from a web-based survey. *Can Pharm J (Ott)* 2017; 150: 407–416.

28. Guénette L, Maheu A, Vanier M-C, et al. Pharmacists practising in family medicine groups: What are their activities and needs? *J Clin Pharm Ther* 2020; 45: 105–114.

29. Gums TH, Carter BL, Milavetz G, et al. Physician-pharmacist collaborative management of asthma in primary care. *Pharmacotherapy* 2014; 34: 1033–1042.

30. Gums T, Uribe L, Vander Weg MW, et al. Pharmacist Intervention for Blood Pressure Control: Medication Intensification and Adherence. *J Am Soc Hypertens* 2015; 9: 569–578.

31. Haag JD, Yost KJ, Kosloski Tarpenning KA, et al. Effect of an Integrated Clinical Pharmacist on the Drivers of Provider Burnout in the Primary Care Setting. *J Am Board Fam Med* 2021; 34: 553–560.

32. Hall LN, Shrader SP, Ragucci KR. Evaluation of compliance with osteoporosis treatment guidelines after initiation of a pharmacist-run osteoporosis service at a family medicine clinic. *Ann Pharmacother* 2009; 43: 1781–1786.

33. Hampson N, Ruane S. The value of pharmacists in general practice: perspectives of general practitioners-an exploratory interview study. *Int J Clin Pharm* 2019; 41: 496–503.

34. Harris IM, Westberg SM, Frakes MJ, et al. Outcomes of medication therapy review in a family medicine clinic. *J Am Pharm Assoc (2003)* 2009; 49: 623–627.

35. Haua R, Harrison J, Aspden T. Pharmacist integration into general practice in New Zealand. *J Prim Health Care* 2019; 11: 159–169.

36. Hazen ACM, de Bont AA, Leendertse AJ, et al. How Clinical Integration of Pharmacists in General Practice has Impact on Medication Therapy Management: A Theory-oriented Evaluation. *Int J Integr Care* 2019; 19: 1.

37. Hazen ACM, Zwart DLM, Poldervaart JM, et al. Non-dispensing pharmacists' actions and solutions of drug therapy problems among elderly polypharmacy patients in primary care. *Fam Pract* 2019; 36: 544–551.

38. Hill D, Marr E, Smith C. Development of Pharmacist Independent Prescribing Clinics to Treat Opioid Analgesic Dependence in NHS Lanarkshire. *Pharmacy (Basel)* 2019; 7: 119.

39. Isetts BJ, Buffington DE, Carter BL, et al. Evaluation of Pharmacists' Work in a Physician-Pharmacist Collaborative Model for the Management of Hypertension. *Pharmacotherapy* 2016; 36: 374–384.

40. James O, Cardwell K, Moriarty F, et al. Pharmacists in general practice: a qualitative process evaluation of the General Practice Pharmacist (GPP) study. *Fam Pract* 2020; 37: 711–718.

41. Jun JK. Establishing Clinical Pharmacy Services With Prescribing Privileges in a Federally Qualified Health Center Primary Care Clinic. *J Pharm Pract* 2018; 31: 434–440.

42. Karampatakis GD, Ryan K, Patel N, et al. Capturing pharmacists' impact in general practice: an e-Delphi study to attempt to reach consensus amongst experts about what activities to record. *BMC Fam Pract* 2019; 20: 126.

43. Karampatakis GD, Ryan K, Patel N, et al. How do pharmacists in English general practices identify their impact? An exploratory qualitative study of measurement problems. *BMC Health Serv Res* 2019; 19: 34.

44. Karampatakis GD, Patel N, Stretch G, et al. Community pharmacy teams' experiences of general practice-based pharmacists: an exploratory qualitative study. *BMC Health Serv Res* 2020; 20: 431.

45. Karampatakis GD, Patel N, Stretch G, et al. Patients' experiences of pharmacists in general practice: an exploratory qualitative study. *BMC Fam Pract* 2021; 22: 48.

46. Kosari S, Deeks LS, Naunton M, et al. Funding pharmacists in general practice: A feasibility study to inform the design of future economic evaluations. *Res Social Adm Pharm* 2021; 17: 1012–1016.

47. Langran T, Nanda N, Bataveljic A, et al. Supporting the management of type 2 diabetes with pharmacist-led reviews: an observational analysis. *BMJ Open* 2017; 7: e013451.

48. MacRae F, Lowrie R, MacLaren A, et al. Pharmacist-led medication review clinics in general practice: the views of Greater Glasgow GPs. *Int J Pharm Pract* 2003; 11: 199–208.

49. Marques I, Gray NJ, Tsoneva J, et al. Pharmacist joint-working with general practices: evaluating the Sheffield Primary Care Pharmacy Programme. A mixed-methods study. *BJGP Open* 2018; 2: bjgpopen18X101611.

50. Maskrey M, Johnson CF, Cormack J, et al. Releasing GP capacity with pharmacy prescribing support and New Ways of Working: a prospective observational cohort study. *Br J Gen Pract* 2018; 68: e735–e742.

51. Moczygemba LR, Alshehri AM, Harlow LD, et al. Comprehensive health management pharmacist-delivered model: impact on healthcare utilization and costs. *Am J Manag Care* 2019; 25: 554–560.

52. Nabhani-Gebara S, Fletcher S, Shamim A, et al. General practice pharmacists in England: Integration, mediation and professional dynamics. *Res Social Adm Pharm* 2020; 16: 17–24.

53. Neilson AR, Bruhn H, Bond CM, et al. Pharmacist-led management of chronic pain in primary care: costs and benefits in a pilot randomised controlled trial. *BMJ Open* 2015; 5: e006874.

54. Nelson PA, Bradley F, Martindale A-M, et al. Skill-mix change in general practice: a qualitative comparison of three 'new' non-medical roles in English primary care. *Br J Gen Pract* 2019; 69: e489–e498.

55. Odenthal DR, Philbrick AM, Harris IM. Successful deprescribing of unnecessary proton pump inhibitors in a primary care clinic. *J Am Pharm Assoc (2003)* 2020; 60: 100–104.

56. Petty DR, Knapp P, Raynor DK, et al. Patients' views of a pharmacist-run medication review clinic in general practice. *Br J Gen Pract* 2003; 53: 607–613.

57. Pottie K, Farrell B, Haydt S, et al. Integrating pharmacists into family practice teams: physicians' perspectives on collaborative care. *Can Fam Physician* 2008; 54: 1714-1717.e5.

58. Pottie K, Haydt S, Farrell B, et al. Pharmacist's identity development within multidisciplinary primary health care teams in Ontario; qualitative results from the IMPACT project. *Res Social Adm Pharm* 2009; 5: 319–326.

59. Prudencio J, Kim M. Diabetes-Related Patient Outcomes through Comprehensive Medication Management Delivered by Clinical Pharmacists in a Rural Family Medicine Clinic. *Pharmacy (Basel)* 2020; 8: 115.

60. Rodgers S, Avery AJ, Meechan D, et al. Controlled trial of pharmacist intervention in general practice: the effect on prescribing costs. *Br J Gen Pract* 1999; 49: 717–720.

61. Rothman R, Malone R, Bryant B, et al. Pharmacist-led, primary care-based disease management improves hemoglobin A1c in high-risk patients with diabetes. *Am J Med Qual* 2003; 18: 51–58.

62. Ryan K, Patel N, Lau WM, et al. Pharmacists in general practice: a qualitative interview case study of stakeholders' experiences in a West London GP federation. *BMC Health Serv Res* 2018; 18: 234.

63. Savickas V, Foreman E, Ladva A, et al. Pharmacy services and role development in UK general practice: a cross-sectional survey. *Int J Pharm Pract* 2021; 29: 37–44.

64. Slazak E, Shaver A, Clark CM, et al. Implementation of a Pharmacist-Led Transitions of Care Program within a Primary Care Practice: A Two-Phase Pilot Study. *Pharmacy (Basel)* 2020; 8: 4.

65. Sloeserwij VM, Hazen ACM, Zwart DLM, et al. Effects of non-dispensing pharmacists integrated in general practice on medication-related hospitalisations. *Br J Clin Pharmacol* 2019; 85: 2321–2331.

66. Sloeserwij VM, Zwart DLM, Hazen ACM, et al. Non-dispensing pharmacist integrated in the primary care team: effect on the quality of physician's prescribing, a non-randomised comparative study. *Int J Clin Pharm* 2020; 42: 1293–1303.

67. Stewart D, Maclure K, Newham R, et al. A cross-sectional survey of the pharmacy workforce in general practice in Scotland. *Fam Pract* 2020; 37: 206–212.

68. Sudeshika T, Naunton M, Peterson GM, et al. Evaluation of General Practice Pharmacists: Study Protocol to Assess Interprofessional Collaboration and Team Effectiveness. *Int J Environ Res Public Health* 2021; 18: 966.

69. Syafhan NF, Al Azzam S, Williams SD, et al. General practitioner practice-based pharmacist input to medicines optimisation in the UK: pragmatic, multicenter, randomised, controlled trial. *J Pharm Policy Pract* 2021; 14: 4.

70. Tan ECK, Stewart K, Elliott RA, et al. Stakeholder experiences with general practice pharmacist services: a qualitative study. *BMJ Open* 2013; 3: e003214.

71. Tan ECK, George J, Stewart K, et al. Improving osteoporosis management in general practice: a pharmacist-led drug use evaluation program. *Drugs Aging* 2014; 31: 703–709.

72. Tan ECK, Stewart K, Elliott RA, et al. Pharmacist consultations in general practice clinics: the Pharmacists in Practice Study (PIPS). *Res Social Adm Pharm* 2014; 10: 623–632.

73. Vande Griend JP, Saseen JJ, Bislip D, et al. An off-site clinical pharmacy service in family medicine: development and 1-year outcomes. *Fam Med* 2014; 46: 348–353.

74. Young S, Bishop L, Twells L, et al. Comparison of pharmacist managed anticoagulation with usual medical care in a family medicine clinic. *BMC Fam Pract* 2011; 12: 88.

75. Zermansky AG, Petty DR, Raynor DK, et al. Randomised controlled trial of clinical medication review by a pharmacist of elderly patients receiving repeat prescriptions in general practice. *BMJ* 2001; 323: 1340-1343.
